# Evidence-based brief interventions targeting acute mental health presentations for children and adolescents: systematic review

**DOI:** 10.1192/bjo.2024.25

**Published:** 2024-04-11

**Authors:** Valsamma Eapen, Brigitte Gerstl, Bright Opoku Ahinkorah, James Rufus John, Patrick Hawker, Thomas P. Nguyen, Febe Brice, Teresa Winata, Michael Bowden

**Affiliations:** Academic Unit of Infant, Child, and Adolescent Psychiatry Services (AUCS), South Western Sydney Local Health District and Ingham Institute, Australia; and Discipline of Psychiatry and Mental Health, University of New South Wales, Australia; Academic Unit of Infant, Child, and Adolescent Psychiatry Services (AUCS), South Western Sydney Local Health District and Ingham Institute, Australia; Discipline of Psychiatry and Mental Health, University of New South Wales, Australia; and School of Public Health, University of Technology Sydney, Australia; Discipline of Psychiatry and Mental Health, University of New South Wales, Australia; Academic Unit of Infant, Child, and Adolescent Psychiatry Services (AUCS), South Western Sydney Local Health District and Ingham Institute, Australia; Discipline of Psychiatry and Mental Health, University of New South Wales, Australia; and Mental Health Team, School of Medicine, Western Sydney University, Australia; Academic Unit of Infant, Child, and Adolescent Psychiatry Services (AUCS), South Western Sydney Local Health District and Ingham Institute, Australia; Academic Unit of Infant, Child, and Adolescent Psychiatry Services (AUCS), South Western Sydney Local Health District and Ingham Institute, Australia; Discipline of Psychiatry and Mental Health, University of New South Wales, Australia; and Infant, Child and Adolescent Mental Health Service (ICAMHS), South Western Sydney Local Health District, Australia; Child and Youth Mental Health, New South Wales Ministry of Health, Australia; Sydney Medical School, University of Sydney, Australia; and Department of Psychological Medicine, Sydney Children's Hospitals Network, Australia

**Keywords:** Mental health services, child and adolescent mental health services, brief intervention, early intervention, crisis intervention

## Abstract

**Background:**

Brief intervention services provide rapid, mobile and flexible short-term delivery of interventions to resolve mental health crises. These interventions may provide an alternative pathway to the emergency department or in-patient psychiatric services for children and young people (CYP), presenting with an acute mental health condition.

**Aims:**

To synthesise evidence on the effectiveness of brief interventions in improving mental health outcomes for CYP (0–17 years) presenting with an acute mental health condition.

**Method:**

A systematic literature search was conducted, and the studies’ methodological quality was assessed. Five databases were searched for peer-reviewed articles between January 2000 and September 2022.

**Results:**

We synthesised 30 articles on the effectiveness of brief interventions in the form of (a) crisis intervention, (b) integrated services, (c) group therapies, (d) individualised therapy, (e) parent–child dyadic therapy, (f) general services, (g) pharmacotherapy, (h) assessment services, (i) safety and risk planning and (j) in-hospital treatment, to improve outcomes for CYP with an acute mental health condition. Among included studies, one study was rated as providing a high level of evidence based on the National Health and Medical Research Council levels of evidence hierarchy scale, which was a crisis intervention showing a reduction in length of stay and return emergency department visits. Other studies, of moderate-quality evidence, described multimodal brief interventions that suggested beneficial effects.

**Conclusions:**

This review provides evidence to substantiate the benefits of brief interventions, in different settings, to reduce the burden of in-patient hospital and readmission rates to the emergency department.

Mental health conditions refer to a wide range of disorders that affect mood, thinking and behaviour in children and young people (CYP), including acute disorders that require immediate attention and intervention. These conditions can have an adverse effect on a child's emotional, social and environmental development, and can lead to long-term adverse effects on their overall health and well-being. Mental health conditions are experienced by approximately 14% of CYP internationally,^1^ which have increased exponentially, particularly in light of the COVID-19 pandemic. According to recent reports, the prevalence of mental health disorders among CYP has increased exponentially, with up to 19% reported for suicidal presentations in Australia.^2^ Suicide is the leading cause of death among young people, with the global suicide rate of 10.5 per 100 000 individuals.^3,4^ According to a 2020 report by United Nations International Children's Emergency Fund (UNICEF), mental health issues among CYP are a growing concern globally. Suicide is the second leading cause of death among young people aged 15–19 years.^5^ The suicide rate has been reported to be 12.0 per 100 000 in the African region, 12.9 per 100 000 in the European region, 13.4 per 100 000 in the South-East Asia region, 11.8 per 100 000 in the USA^6^ and 10.4 per 100 000 in Canada.^7^ The lowest reported suicide rate is 4.3 per 100 000 individuals in the Eastern Mediterranean region.^4^

A mental health crisis in CYP can arise when they encounter an experience or event that exceeds their and/or their family's capacity to manage their mental health distress, resulting in a significant impairment of their ability to function and requiring urgent medical attention.^8,9^ Validated screening tools are available to identify CYP who require brief interventions to address acute mental health crises. These measurement tools, rigorously validated through scientific scrutiny and empirical evidence, are designed to adhere to stringent psychometric standards. By applying established criteria, these screening tools systematically evaluate the mental health status of CYP, enabling healthcare professionals to discern the severity and urgency of intervention required. The use of validated screening tools ensures the precision and reliability of the assessment process, facilitating the timely and targeted implementation of brief interventions for CYP experiencing acute mental health crises.^10^

In situations where risks are higher and the criterion is met, in-patient treatment may be necessary, and many CYP who meet the threshold could benefit from brief interventions.^8,9^ However, in-patient hospital admissions can place a significant burden on both CYP and their caregivers because of dislocation from family, friends and support networks, with readmission occurrences heightening this situation. Our recent work in Australia has found a substantial increase in CYP presenting to the emergency department^8^ and suicide-related ambulance calls^9^ during the COVID-19 pandemic, indicating the need to efficiently respond to an acute mental health condition/presentation/crisis and fill service gaps.^11^ Therefore, safe and effective brief interventions have been proposed to improve the efficiency and effectiveness of crisis care in a timely, safe and sensitive manner, accounting for the complex, multifaceted needs of consumers.

## Aim of the review

The findings of our systematic review are timely and build upon previous reviews reporting outcomes on brief interventions by Otis et al,^12^ Clisu et al^13^ and Newton et al.^14^ These studies collectively suggest that brief interventions have a beneficial impact on reducing readmission rates to the emergency department, which can reduce the burden on emergency departments and improve outcomes for CYP with mental health concerns. In alignment with these previous studies, which highlight the positive impact of brief interventions on reducing readmission rates to emergency departments and enhancing outcomes, our study hypothesises that strategically tailored brief interventions for mental health crises, incorporating established screening procedures and administered in out-patient settings (excluding emergency departments), possess the potential to significantly reduce reliance on emergency department assessments, re-evaluations or in-patient admissions among CYP aged 0–17 years. This hypothesis serves to guide this review, aligning with our aim to contribute essential insights that advance crisis care strategies in the realm of CYP mental health.

## Method

For this review, we followed the guidelines outlined in the Preferred Reporting Items for Systematic Reviews and Meta-Analyses (PRISMA) statement.^15^ The protocol was registered with the International Prospective Register of Systematic Reviews (PROSPERO; registration number CRD42022323324). Ethical approval was not required as this is a systematic review of published data.

### Search strategy and study selection

This systematic literature review utilised five electronic databases (PubMed, PsycINFO via ProQuest, Web of Science via Clarivate, EMBASE and Cochrane Library) to extract studies reporting on the effectiveness of interventions for acute mental health presentations of CYP aged 0–17 years. We also searched cross-references for further articles. Peer-reviewed studies published in the English language were searched over the past 22 years (1 January 2000 to 30 March 2022). We included primary literature evaluating the effectiveness of brief interventions for CYP aged 0–17 years who presented with an acute mental health concern. The full search strategy is included in Supplementary Table 1 available at https://doi.org/10.1192/bjo.2024.25. Search results were de-duplicated in Endnote X9 for Windows (Clarivate Analytics, Berkeley, California, USA; see https://endnote.com/) and again in Rayyan software for Windows for systematic reviews (Rayyan Systems, Cambridge, Massachusetts, USA; see www.rayyan.ai).^16^ A data collection tool was used to screen each paper for inclusion and exclusion eligibility for the review.

### Data extraction and screening

Four reviewers (F.B., B.G., P.H., J.R.J.) performed the initial title and abstract screening with the data collection tool. Two reviewers (F.B., P.H.) independently conducted full-text screening and compared results. At each stage of the selection process, a fifth reviewer (T.W.) was available to resolve or moderate any disagreements on the included articles. Four reviewers (F.B., B.G., P.H., B.O.A.) performed data extraction for the included articles. In parallel, these reviewers extracted data from the full-text reports with Rayyan software, a web-based systematic review application.^16^ This was used to extract study designs, country, the size of the sample, different characteristics of the study population (including age, ethnicity, gender and diagnoses), type of intervention, characteristics of the control group and the outcomes of interest.

### Eligibility criteria

Studies were included if (a) the study reported on the effectiveness of a brief intervention (defined below) targeted at improving mental health outcomes for CYP presenting with an acute mental health condition/presentation/crisis; (b) participants’ age ranged from 0 to 17 years, or where the age range was not reported, the mean age of the participant was <18 years; (c) participants presented with an acute mental health condition/presentation/crisis and (d) studies were published in a peer-reviewed journal in English.

Studies were excluded if (a) participant age criteria were not met (participants >18 years of age), (b) participants were not presenting/attending the intervention for the treatment of an acute mental health condition, (c) the study was a quantitative meta-analysis of published literature and (d) the study was not published in a peer-reviewed journal in English.

### Brief intervention services

We defined the term ‘acute mental health crisis’ as a situation in which CYP experience a sudden and severe deterioration in their mental health. This deterioration is often accompanied by significant distress and impairment in their ability to function. In contrast, we will use the term ‘psychiatric emergency’ to refer to situations where there is an immediate risk to the safety or well-being of the CYP or others because of their mental health condition. In this review, we defined ‘brief interventions’ as an intervention that consisted of three or fewer visits to a service or ≤8 weeks of intervention programme duration. The narrative synthesis followed guidance for systematic reviews to assist with evaluating the evidence-based effectiveness of each intervention, such as what interventions (mechanisms) were effective (outcomes) and the type of setting and location (context) where the intervention took place.^17^

Details describing quality assessment, data extraction and risk of bias can be found in Supplementary Appendices 1 and 2.

### Statistical analysis

Previously reported data from various studies were synthesised. Given the narrative synthesis nature of our work, we focused on the qualitative integration of findings rather than employing quantitative statistical methods. Our approach involved an examination of the reported outcomes, utilising frequencies and percentages where appropriate, to convey the distribution of data. This facilitated a nuanced exploration of the collective evidence, aligning with the synthesis objectives of this review.

## Results

**Fig. 1:**
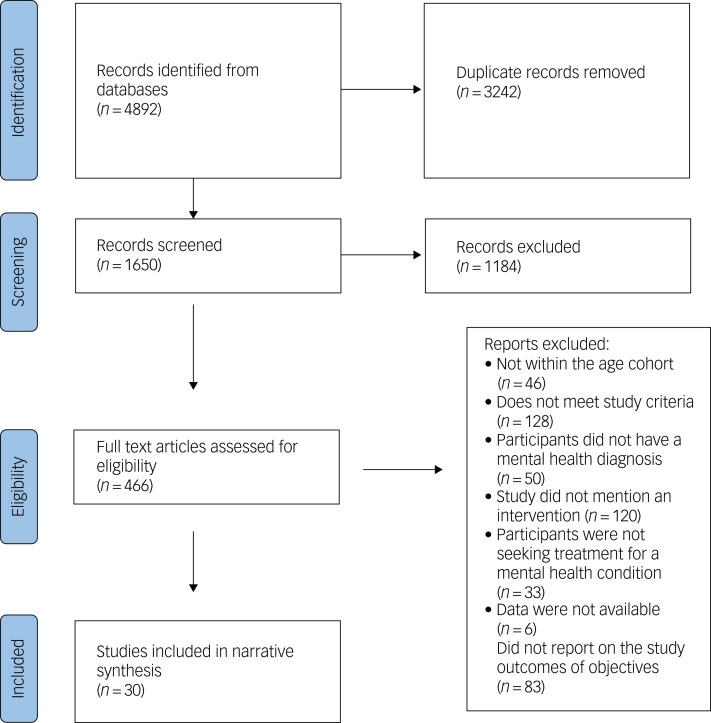
Preferred Reporting Items for Systematic Reviews and Meta-Analyses (PRISMA) flow diagram of study selection process chart.

Our initial search yielded 4892 results, of which 3242 were duplicates, resulting in 1650 articles that were eligible for screening. After title and abstract screening, 436 potentially eligible studies were assessed for eligibility, and 30 studies met the eligibility criteria. Figure 1 illustrates the study selection process, using the PRISMA flow diagram.

Table 1 summarises characteristics of the included studies. Studies were conducted in different geographical regions; 19 studies were from the USA (*n* = 19),^18,37^ and the rest were from Canada (*n* = 7),^38,44^ England (*n* = 2)^45,46^ and Australia (*n* = 1).^47^ Most of the studies were cohort studies (*n* = 19),^18,21,25,27,28,31,39,42,44,48^ followed by pre and post studies (*n* = 10) and one non-randomised controlled trial (*n* = 1).

**Table 1:** Characteristics of participants who received brief interventions

	Intervention, *n* (%)	Control, *n* (%)
Number of studies	30	6
Participants	20 395 (70)	8570 (30)
Gender		
Female	9267 (49)	1983 (53)
Male	9673 (51)	1791 (47)
Age		
Mean age (years) (s.d.)	13.9 (2.4)	13.4 (3.0)
Age group (years)		
0–2	Not available	Not available
3–5	1 (7)	Not available
6–11	Not available	1 (17)
12–17	29 (93)	5 (83)
Ethnicity		
White	4662 (59.5)	1006 (66.0)
Black	2045 (26.1)	320 (21.0)
Latino/Hispanic	945 (11.9)	143 (9.4)
Asian	13 (0.2)	4 (0.3)
First Nations/ATSI	Not available	Not available
Mixed/other	178 (2.3)	52 (3.4)

Brief intervention was defined as three or fewer visits to a service or ≤8 weeks of intervention programme duration. ATSI, Aboriginal and Torres-Strait Islander.

### Methodological quality of the studies

Table 2 provides a comprehensive overview of each of the included studies, with detailed descriptions of the studies, National Health and Medical Research Council (NHMRC) levels of evidence and risk of bias, interventions examined and the results reported. Among the included studies, there was one study that was rated as high quality or provided a high level of evidence based on the NHMRC levels of evidence hierarchy scale (level of evidence I or II),^40^ 10 studies were rated as providing moderate evidence (level of evidence III-2: comparative studies with controls such as cohort studies)^8,19,21,27,33,34,37,42,44^ and 19 studies were of lower-quality evidence (level of evidence III-3: cohort studies without a comparison group; or level of evidence IV: pre and post studies).^18,22,26,29–32,35,36,38,39,41,45,47^ There was considerable variability between study methodologies and 30% (*n* = 9/30 studies) of studies compared outcomes with a comparison group^19,21,27,28,33,37,42,44^ (see Table 2).

**Table 2:** Characteristics of brief intervention studies (*N* = 30 studies)

Author	Year	Country	NHMRC level of evidence and risk of bias (high, moderate or low)	Study design	Sample size	Mean age	Gender	Acute/crisis mental health symptoms	Intervention strategies	Acute treatment setting	Outcomes
Adrian and Smith^a^ (Group 1: HSO)^45^	2015	England	Evidence: low (level: IV) Risk of bias: moderate	Pre- and post-study	INT *n* = 287	INT 16.3 years	INT Male: 179/287 Female: 108/287	INT Emergency symptoms (not specified) (*n* = 184/287) Psychosis disorders (*n* = 33/287) Autism spectrum disorder (*n* = 6/287) Depression and mood disorders (*n* = 61/287) Suicidal behaviours (*n* = 20/287) Eating disorders (*n* = 5/287)	CBT General services Group therapy Family therapy Pharmacotherapy or medication management Integrated services	AAOT is an out-patient community-based service that provides intensive community treatment	Decrease in mean HoNOSCA scores post-treatment (intensive home support only treatment) (23.72 *v.* 14.95). Improvement in mean CGAS scores post-treatment (45.58 *v.* 62.40)
Adrian and Smith^a^ (Group 2: ARC)^45^	2015	England	Evidence: low (level: IV) Risk of bias: moderate	Pre- and post-study	INT *n* = 211	INT 16.5 years	INT Male: 45/211 Female: 166/211	INT Emergency symptoms (not specified) (*n* = 105/211) Psychosis disorders (*n* = 27/211) Autism spectrum disorders (*n* = 1/211) Depression and mood disorders (*n* = 33/211) Anxiety (*n* = 24/ 211) Self-harm/suicidal ideation (*n* = 14/211) Eating disorder (*n* = 2/211)	CBT General services Family therapy Pharmacotherapy or medication management Integrated services In-hospital treatment	AAOT is an out-patient community-based service providing intensive community treatment	Decrease in mean HoNOSCA scores post-treatment (adolescent assertive outreach team support as well as in-patient care) (22.45 *v.* 14.40) Improvement in mean CGAS scores post-treatment (46.22 *v.* 62.00)
Aupont et al^18^	2013	USA	Evidence: low (level: IV) Risk of bias: moderate	Cohort study	INT *n* = 329	INT 12.3 years	INT Male: 188/329 Female: 141/329	INT ADHD (*n* = 128/329) Conduct disorder or oppositional defiant disorders (*n* = 23/329) Depression and mood disorders (102/329) Anxiety (*n* = 43/329) Developmental disorder (*n* = 13/329) Emergency symptoms (not specified) (*n* = 20/329)	General services Pharmacotherapy or medication management	Primary care setting, specifically in primary care practices that provided paediatric care	CYP with major depression or anxiety disorders were less likely to return to primary care paediatricians compared with CYP with ADHD following the intervention (Targeted Child Psychiatric Services programme). Families widely accepted paediatricians’ recommendations for referral to child psychiatrists. 28.7% CYP returned to their referring paediatricians for follow-up management of their mental disorder following the intervention. Most CYP (*n* = 52) returned to their referring paediatrician after a single evaluation visit. For ADHD (*n* = 129), anxiety (*n* = 43) and major depressive disorder (*n* = 102), the return rates were 48.8, 27.9 and 5.9%, respectively. For CYP with developmental delay, conduct, eating and other disorders, return rates to primary care were, on average, 20.0%, with a range from 8.3% (conduct disorders) to 36.8% (eating disorders)
Carlisle et al^44^	2012	Canada	Evidence: moderate (level: III-2) Risk of bias: moderate	Cohort	INT CYP with aftercare: *n* = 1502/3004 CNT CYP without aftercare: *n* = 1502/3004	INT Mean age: 16.9 years CNT Mean age:16.9 years	INT Males: 872/1502 Females: 630/1502 CNT Males: 602/1502 Females: 900/1502	INT Adjustment disorder: 174/1502 Anxiety: 92/1502 Behavioural disorders: 51/1502 Eating disorders: 60/1502 Depression and mood disorder: 575/1502 Bipolar disorder: 57/1502 Psychosis: 141/1502 Self-harm/suicidal ideation: 218/1502 Substance misuse: 91/1502 Emergency symptoms (not specified): 63/1502 CNT Adjustment: disorder: 199/1502 Anxiety: 97/1502 Behavioural disorders: 40/1502 Eating disorders: 49/1502 Depression and mood disorder: 590/1502 Bipolar disorder: 38/1502 Psychosis: 123/1502 Self-harm/suicidal ideation: 230/1502 Substance misuse: 69/1502 Emergency symptoms (not specified): 66/1502	In-hospital Integrated services	Mental health services for CYP provided in a variety of settings, including hospitals and clinics staffed by psychiatrists, general practitioners, paediatricians, nurses, social workers and counsellors	Adolescents with aftercare (primary care physician or psychiatrist as referral from in-patient setting: 30-day follow-up or out-patient clinic) had more readmissions at 1 year follow-up (18.8% *v.* 14.8%), had shorter mean time to first readmission (347.68 days *v.* 357.37 days) and were 38% more likely to have readmission
Casher et al^19^	2022	USA	Evidence: moderate (level: III-2) Risk of bias: moderate	Cohort	INT *n* = 71 CNT *n* = 71	INT mean age not reported (age range: 3–19 years) CNT mean age not reported (age range: 3–19 years)	INT Male: 32/71 Female: 39/71 CNT: Male: 32/71 Female:39/71	INT Emergency symptoms (not specified) (*n* = 71/71) CNT Emergency symptoms (not specified) (*n* = 71/71)	Behavioural health Integrated services Wellness therapy Assessment services General services Safety planning Substance/drug/alcohol counselling	The intervention was delivered in a paediatric emergency department	There was no significant difference among groups in return rates within 90 days among intervention versus nurse assessment or psychiatric emergency service patients (25% *v.* 23% *v.* 13%)
Grimes et al^20^	2018	USA	Evidence: moderate (level: III-2) Risk of bias: moderate	Cohort	INT: *n* = 29 CNT: *n* = 157	INT 12.5 years (age range: 4–19 years) CNT 8.9 years (age range: 4–19 years)	INT Male: 20/29 Female: 9/29 CNT: Male: 95/157 Female: 62/157	INT Emergency symptoms (not specified) (*n* = 29/29) CNT Emergency symptoms (not specified) (*n* = 157/157)	Integrated services Individualised treatment or therapy General services	Mental health service use outcomes for children referred by paediatricians for out-patient child psychiatry evaluation within an urban safety-net hospital system	The intervention group (an integrated collaborative-practice model that combined clinical care within paediatrics and community-based parent support from family support specialists of CYP were significantly more likely to engage in treatment than control CYP (79% *v.* 48%) Intervention group were four times more likely to access treatment than the control group (control group received treatment as usual) (92% *v.* 75%). 80% of CYP in intervention who had completed psychiatric evaluation would be expected to engage in further care compared with 49% of CYP receiving treatment as usual. 74% of CYP in the intervention group receiving a referral for a psychiatric evaluation completed the referral and engaged in recommended follow-up treatment compared with about 37% of youths receiving a referral under usual care conditions
Cheng et al^43^	2017	Canada	Evidence: moderate (level: III-2) Risk of bias: moderate	Cohort	INT *n* = 6558 CYP with aftercare: *n* = 4577/6558 CNT CYP without aftercare: *n* = 1981/6558	INT No mean age reported (age range: 5–24 years) CNT No mean age reported (age range: 5–24 years)	INT Males: 2307/4577 Females: 2270/4577 CNT Males: 1038/1981 Females: 943/1981	INT Substance use disorder: 949/4577 Psychosis: 1297/4577 Depression or mood disorder: 2648/4577 Anxiety disorder: 808/4577 Bipolar disorder: 233/4577 Emergency symptoms (not specified): 3305/4577 CNT Substance use disorder: 947/1981 Psychosis: 325/1981 Depression or mood disorder: 911/1981 Anxiety disorder: 305/1981 Bipolar disorder 73/1981 Emergency symptoms (not specified): 1645/1981	In-hospital Assessment Occupational therapy	In-hospital setting	Aftercare was associated with a 32% reduction in readmission
Cummings et al^25^	2020	USA	Evidence: low (level: III-3) Risk of bias: high	Cohort	INT *n* = 41	INT 15.1 years (range:5–24 years)	INT Male: 34/41 Female: 7/41	INT Autism (*n* = 41/41)	Integrated services Pharmacotherapy and medication management	Psychiatric emergency department and services provided in the community	Clinical and Family Distress Scale scores indicated significant improvements for CYP and caretakers. There was a 33% reduction in time spent in the emergency department, and LOS decreased up to 77% from pre- to postintervention. There was a 6% reduction in visits to the emergency department postintervention
Gillig et al^36^	2004	USA	Evidence: low (level: III-3) Risk of bias: high	Cohort	INT *n* = 48	INT 16.5 years (age range: 12–18 years)	INT Male: 22/48 Female: 26/48	INT Depression and mood disorder (*n* = 32/48) Conduct disorder (*n* = 8/48) Psychosis (*n* = 7/48) Anxiety (*n* = 3/48) Eating disorders (*n* = 2/48) Alcohol and drug misuse (1/48)	Crisis intervention (emergency evaluation plus review) Individual therapy	Emergency department and in-hospital setting	10% of CYP seen in the emergency department were admitted to hospital after the evaluation was received, no patients were admitted to hospital in the month following the evaluation and 4.2% patients were admitted to hospital 6 months later
Greenham and Bisnaire^38^	2008	Canada	Evidence: low (level: III-3) Risk of bias: moderate	Cohort	INT *n* = 211	INT 14.9 years (age range: 10–17 years)	INT Male: 67/211 Female: 144/211	INT Depression and mood disorder (*n* = 97/211) Adjustment disorder (*n* = 19/211) Psychosis (*n* = 19/211) Behaviour disorders (*n* = 15/211) Emergency symptoms (not specified) (*n* = 11/211) Self-harm/suicidal ideation (*n* = 186/211) Anxiety (*n* = 44/211) Eating disorders (*n* = 17/211)	Integrated services In-hospital treatment Crisis intervention Assessment Transitional care services	In-patient psychiatric and mental health services	LOS was consistent with the model of care and differed for youth receiving only crisis (4 days) versus crisis plus assessment services (13 days). Youth referred for in-patient transitional care had longer LOS for crisis/assessment services (19 days). Crisis and assessment CYP were more likely than CYP transferred for transitional care to be admitted as an in-patient. The assessment group reported higher levels of emotional and behavioural concerns on the YSR than other groups; significant only for internalising problems, and anxiety/depression assessment youth had significantly higher scores than crisis youth. All groups had clinically elevated scores on the internalising problems and anxiety/depression scales. Parent/guardian reports of youth's emotional and behavioural functioning on the CBCL were higher for the assessment group compared with other groups. 80% of CYP in each group showed reliable improvement in total acuity level. Most of the youth in each group improved on the four CAPI subscales. Systems support: less than 50% of CYP in the crisis and assessment groups showed improvement. Compared with other groups, more transition youth showed a reliable increase in total acuity level at the time of transfer (15% *v.* 9 and 6%)
Gusella et al^42^	2017	Canada	Evidence: moderate (level: III-2) Risk of bias: moderate	Cohort	INT *n* = 46 non-specific therapy group: *n* = 14/36; family therapy group: *n* = 32/46	INT Non-specific therapy group*:* mean age: 13.07 years (range: 9–15 years) Family therapy group: mean age: 14.36 years (range: 9–15 years)	Non-specific therapy group: Male: 0/14 Female: 14/14 Family therapy group: Male: 3/32 Female: 29/32	Eating disorders (*n* = 46)	Outreach Group therapy Pharmacotherapy and medication management General services In-hospital	In-patient hospital stay and out-patient care provided by a tertiary healthcare centre	Reduced readmissions following family therapy (34.4% *v.* 71.4%) and reduction in LOS (50 *v.* 19.1 days) following family therapy.
Hasken et al^26^	2022	USA	Evidence: low (level: IV) Risk of bias: high	Pre- and post-study	INT *n* = 317 (before psychiatric unit opened: 91/317; after psychiatric unit opened: 226/317)	INT Mean age was 12.9 years (age range: 2–22 years)	INT Before Male: 41/91 Female: 50/91 After Male: 101/226 Female: 126/226	Emergency symptoms (not specified) (*n* = 317/317)	General services Assessment services In-hospital	Urban tertiary care paediatric emergency department at a large tertiary care centre	Emergency department admissions reduced 22.2–8.5% following the intervention with an admission to the psychiatric crisis unit in the emergency department (staffed by psychiatry team with psychological therapies). LOS increased from 363 to 418 min with an admission to in-patient medical ward or transferred to a psychiatric unit
Holder et al^21^	2017	USA	Evidence: moderate (level: III-2) Risk of bias: high	Cohort	INT *n* = 1983 CNT *n* = 1237	INT 14.3 years (age range: 5–18 years) CNT 14.9 years (age range: 5–18 years)	INT Male: 1045/1983 Female: 938/1983 CNT: Male: 665/1237 Female: 572/1237	INT Emergency symptoms (not specified) (*n* = 1983/1983) CNT Emergency symptoms (not specified) (*n* = 1237/1237)	Integrated services	Paediatric emergency department	LOS in emergency department was reduced from 14.7 to 12.1 h following the intervention (8 h daily psychiatrist, and a referral to the psychiatric unit). Admissions decreased following the intervention (from 17 to 1%)
Huryk et al^34^	2021	USA	Evidence: moderate (level: III-2) Risk of bias: moderate	Cohort	INT *n* = 326	Mean age in-patient hospital group: 16 years Mean age family therapy: 15.7 years (age range: 8–21 years)	–	Eating disorders: (*n* = 326/326)	Wellness therapy Group therapy In-hospital Individual sessions	Partial hospital stay programme	Rates of readmission were significantly lower for those who received care during the implementation of FBT intervention compared with treatment as usual (3.6% *v.* 12.2%)
Ishikawa et al^40^	2021	Canada	Evidence: high (level: III-1) Risk of bias: moderate	Non-randomised controlled trial	INT *n* = 3467/6576 CNT *N* = 3109/6576	INT 13.1 years (age range: 0–17 years) CNT 13.9 years (age range: 0–17 years)	–	INT Emergency symptoms (not specified) (*n* = 3220)	Integrated services Crisis intervention	Emergency department managing paediatric mental health presentations	A reduction in 30-day readmission LOS reduced by 85.3 min and a 15.2% reduction in 30-day return visits by CYP presenting to emergency department
Kells et al^33^	2017	USA	Evidence: moderate (level: III-2) Risk of bias: moderate	Cohort	INT *n* = 56 CNT *n* = 52	INT Mean age was 17.4 years CNT Mean age was 14.8 years	-	Eating disorders (*n* = 108)	In-hospital General service	Medical hospital admission for restrictive eating disorders	LOS was 3 days shorter for the intervention group (psychological support: 3 × 30-min meal supervision per day per person) compared with the control group (20 days *v.* 23 days)
Knapp et al^22^	2007	USA	Evidence: low (level: III-3)	Pre- and post-test	INT *n* = 388	INT mean age not reported (age range: 0–5 years)	INT Male: 238/388 Female: 150/388	INT Anxiety (*n* = 62/388) Behaviour disorders (*n* = 89/388) Adjustment disorder (*n* = 89/388) Affect or reactive attachment disorder (*n* = 70/388) Emergency symptoms (not specified) (*n* = 66/388)	Family therapy Parent–child dyad therapy	Eight California county mental health systems, where mental health providers were trained to provide mental health screening and relationship-based intervention to expand services for children	After intervention, Mental Health Screening and Risk Assessment scores were significantly lower. GAF scores and Parent-Infant Relationship Global Assessment Scale scores increased significantly
Mahajan et al^27^	2007	USA	Evidence: moderate (level: III-2) Risk of bias: high	Cohort	INT *n* = 531 CNT *n* = 500	INT/CNT mean age was 12.5 years (age range: 0–19 years)	INT/CNT Male: 603/1031 Female: 428/1031	Emergency symptoms (not specified) (*n* = 3220)	Crisis intervention	Inner-city paediatric emergency department	LOS reduced (259.49 min *v.* 216.39 min) after initiation of the programme (intervention)
Martin et al^23^	2013	USA	Evidence: low (level: IV) Risk of bias: moderate	Pre- and post-study	INT *n* = 110	INT 4.2 years (age range: 2–5 years)	INT Male: 77/110 Female: 33/110	INT Behavioural disorders (*n* = 110/110)	Safety planning Family therapy Pharmacotherapy or medication management Outreach	Specialised, family focused psychiatric partial hospital stay programme or preschool-aged children with severe psychopathology	CBCL revealed that CYP symptoms had significantly decreased from the time of admission for externalising problems and total problem. Mean scores on the CBCL for both severity on externalising and internalising problems also decreased from admission to discharge. The normative comparisons and the Reliable Change Index showed that 41% of CYP were functioning in the normative (nonclinical) range on discharge for externalising behaviour problems and 29% were in the nonclinical range for internalising problems at discharge. 56 and 32% of children demonstrated clinically meaningful change relative to externalising and internalising behaviour problems, respectively, at discharge
McBee-Strayer et al^24^	2019	USA	Evidence: low (level: IV) Risk of bias: moderate	Pre- and post-study	INT *n* = 50	INT 15.1 years (age range: 12–17 years)	INT Male: 7/50 Female: 43/50	INT Self-harm/suicidal ideation (*n* = 50/50)	CBT Family therapy Integrated services	Intensive crisis stabilisation unit that provides intervention (ICI), a cognitive–behavioural family-centred treatment for adolescents with suicidal ideation and/or attempts	Follow-up data at 3 months showed that the mean Suicidal Ideation Questionnaire- Junior score improved by 34.2 points compared with baseline following the intervention (Intensive Crisis Intervention). Significant improvements in functioning, high rates of consumer satisfaction and readiness for care transition upon discharge were also reported
McDowell et al^35^	2020	USA	Evidence: low (level: III-3) Risk of bias: high	Cohort	INT *n* = 81	No mean age reported (age range: 9–17 years)	INT Male: 17/81 Female: 64/81	Anxiety (*n* = 81/81)	Wellness therapy Integrated services Crisis intervention Family therapy Group therapy	Behavioural healthcare system that provides crisis management services for acutely anxious adolescents who require higher intensity services	30-day (9.5%) and 90-day readmissions (15.6%) reduced following the intervention of psychoeducation (6 × 1 h per week, inclusive of mindfulness) delivered by two mental health clinicians and a yoga teacher
Morris et al^39^	2009	Canada	Evidence: low (level: IV) Risk of bias: high	Cohort	INT *n* = 56	INT 14.3 years (age range: 9–17 years)	INT Male: 36/56 Female: 20/56	INT Psychosis (*n* = 56/56) Behaviour disorders (*n* = 10/56)	CBT General services Pharmacotherapy or medication management Assessment services Group therapy Occupational therapy In-hospital treatment	Out-patient Early Psychosis Intervention service	Readmission and hospital admission rates following the Early Psychosis Intervention (including psychoeducation) service decreased. Among discharged patients with psychosis, the mean CGAS scores improved from initial psychiatric assessment to discharge (53.7 to 56.3). The mean CGAS at admission for patients who dropped out also improved from a mean score of 51.7 at last visit to 52.5 at a subsequent visit
Parast et al^28^	2018	USA	Evidence: low (level: III-3) Risk of bias: moderate	Cohort	INT Total = 378 (emergency department: *n* = 194; in-patient *n* = 184)	INT mean age not reported (age range: 5–17 years)	INT Male: 132/378 Female: 246/378	INT Self-harm/suicidal ideation (*n* = 378)	General services	Hospital-based care for suicidal youth	Admissions reduced following the intervention of psychoeducation (risk prevention delivered in emergency department and in-patient setting by emergency care team) (32.8–24.5%)
Parker et al^41^	2003	Canada	Evidence: low (level: IV) Risk of bias: high	Pre- and post-study	INT *n* = 340	INT mean age was 13.2 years (age range: 0–19 years)	INT Male: 167/340 Female: 173/340	INT Emergency symptoms (not specified) (*n* = 340/340)	Crisis intervention	Accident and emergency department or urgent consultation clinic of the Child and Adolescent Psychiatry Division	Admissions to emergency department reduced following psychiatric triage and crisis support team (emergency department assessment and urgent care referrals) from 6.3 to 2.3%
Reliford and Adebanjo^29^	2019	USA	Evidence: low (level: IV) Risk of bias: high	Pre- and post-study	INT *n* = 35	INT mean age not reported (age range: 3–18 years)	-	INT Emergency symptoms (not specified) (*n* = 35/35)	Assessment services	Paediatric emergency room where child psychiatry patients were evaluated and followed up with telepsychiatry	Three months LOS in emergency department reduced for non-hospitalised patients following a telepsychiatry intervention (alternative to face-to-face consultation) (285 *v.* 193 h)
Rogers et al^32^	2015	USA	Evidence: low (level: III-3) Risk of bias: high	Cohort	INT *n* = 3582 (Pre-intervention: 1719 Post-intervention: 1863)	INT Pre-intervention mean age: 12.9 years Post-intervention mean age: 13.2 years	INT Pre-intervention Male: 877/1719 Female: 842/1719 Post-intervention Male: 913/1863 Female: 950/1863	Depression and mood disorder (data not specified) Bipolar (data not specified)	Crisis intervention	Paediatric emergency department	Emergency department LOS decreased (from 14.7 to 12.1 h) following the intervention, which consisted of a psychiatric crisis unit (six-bed unit, MDT assessment, intensive care and stabilisation, psychiatric nursing team)
Schley et al^47^	2012	Australia	Evidence: low (level: IV) Risk of bias: high	Pre- and post-study	INT *n* = 44	INT mean age not reported (age range: 14–25 years)	INT Male: 9/44 Female: 35/44	INT Emergency symptoms (not specified) (*n* = 44/44) Self-harm/suicidal ideation (*n* = 44/44) Aggression (*n* = 40/44) Substance misuse (*n* = 26/44)	Integrated services	Intensive Mobile Youth Outreach Service	Good client engagement was achieved after the intervention, which comprised an intensive mobile youth outreach service. Mean engagement scores improved following the outreach intervention at discharge. Lower risk of CYP hostility to themselves decreased following the intervention and the overall level of functioning and well-being improved
Sclare et al^46^	2015	England	Evidence: low (level: IV) Risk of bias: moderate	Pre- and post-study	INT *n* = 31	INT 16.7 years age range: 16–18 years)	INT Male: 11/31 Female: 20/31	INT Behaviour disorders (*n* = 31/31)	CBT	Not an acute setting	Pre- and post- intervention (the intervention included a 1-day CBT workshop), 64.5% had significant improvements on all outcome measures (SCARED, MFQ and RSES). A decrease in mean anxiety scores on the SCARED tool post-workshop (26.85 *v.* 20.40). A decrease in the MFQ clinical outcome scores pre- to post-workshop (19.85 *v.* 10.95) and an increase in the Rosenberg Self-Esteem Scale RSES scores (18.50 *v.* 20.20)
Sheridan et al^30^	2015	USA	Evidence: low (level: IV) Risk of bias: moderate	Pre- and post-study	INT *n* = 212 (Pre-intervention: 83 Post-intervention: 129)	INT Pre-intervention mean age: 13.5 years Post-intervention mean age: 13.9 years	INT Pre-intervention Male: 48/83 Female:35/83 Post-intervention: Male: 45/129 Female: 55/129	INT Pre-intervention: Self-harm/suicidal ideation: 41/83 Depression and mood disorder: 49/83 Anxiety:7/83 Conduct disorder:9/83 Emergency symptoms (not specified): 19/83 Post-intervention Self-harm/suicidal ideation: 75/129 Depression and mood disorder Anxiety: 11/129 Conduct disorder:11/129 Emergency symptoms (not specified): 39/129	Crisis intervention	Paediatric emergency department	LOS in the emergency department decreased by 27% after the intervention (intervention included a paediatric psychiatric consultation). Admissions reduced by 45% after implementation of the intervention. However, suicidality increase in the postintervention period
Uspal et al^31^	2016	USA	Evidence: low (level: III-3) Risk of bias: high	Cohort	INT *n* = 1640 (Pre-intervention: 738 Post-intervention: 902)	INT Pre-intervention mean age: 13.5 years Post-intervention mean age: 13.8 years	INT Pre-intervention Male: 406/738 Female: 332/738 Post-intervention Male: 442/902 Female: 500/902	Emergency symptoms (not specified): 1640/1640	Crisis intervention	Tertiary care children's hospital's emergency department	Mean LOS in emergency department reduced from 332 to 244 min following the intervention, which included a dedicated psychiatric triage and treatment team (this included psychiatric nurse or social worker, and a practitioner) and 24/7, individual and family psychoeducation, discharge planning.
Wharff et al^37^	2012	USA	Evidence: medium (level: III-2) Risk of bias: moderate	Cohort	INT *n* = 100 CNT *n* = 150	INT mean age was 15.6 years (age range: 13–18 years) CNT No mean age reported	INT Male: 24/100 Female: 76/100 CNT Male: 24/150 Female: 39/150	INT Depression or mood disorder:77/100 Bipolar disorder: 5/100 Anxiety: 8/100 Emergency symptoms (not specified): 11/100 CNT Depression or mood disorder:107/150 Bipolar disorder: 10/150 Anxiety: 10/150 Emergency symptoms (not specified): 23/150	Group therapy	Large, urban paediatric emergency room	Significant decrease in emergency department admission rate from pre- FBCI in the emergency room (single session) (55%) to post-FBCI (35%)

NHMRC, National Health and Medical Research Council; HSO, patients who were not admitted to hospital; INT, intervention group; CBT, cognitive–behavioural therapy; AAOT, adolescent assertive outreach team; HoNOSCA, Health of the Nation Outcome Scales; CGAS, Children's Global Assessment Scale; ARC, adolescent resource centre; ADHD, attention-deficit hyperactivity disorder; CYP, children and young people; CNT, control group; LOS, length of stay; YSR, youth self-report; CBCL, Child Behavior Checklist; CAPI, Childhood Acuity of Psychiatric Illness Scale; FBT, family-based treatment; GAF, Global Assessment of Functioning; ICI, intensive crisis intervention; MDT, multidisciplinary team; SCARED, Screen for Child Anxiety Related Emotional Disorders; MFQ, Mood and Feelings Questionnaire; RSES, Rosenberg Self-Esteem Scale; FBCI, family-based crisis intervention.

a. Two interventions incorporated into the same study.

### Effect of interventions on mental health symptoms

We narratively synthesised intervention types into the following categories: crisis intervention, integrated services, group therapies, individualised therapy, parent–child dyadic therapy, general services, pharmacotherapy, assessment services, safety and risk planning, and in-hospital treatment (see Table 2).

#### Assessment services

Five studies (17%) evaluated assessment services, including motivational interviewing, neuropsychology assessment, telepsychiatry and interdisciplinary assessment, targeting CYP (age range: 3–17 years) with psychosis and behavioural disorders.^19,26,38,39^ Although information about mental health disorders for younger children accessing assessment services was not specified, Reliford and Adebanjo's study^29^ demonstrated low-level evidence that on-call telepsychiatry consultations in a non-hospitalised intervention programme significantly reduced the total monthly length of stay (LOS) during a 6-month study period (from 285 to 193 h) compared with prior months. Non-hospitalised patients also experienced a significant reduction in monthly LOS, decreasing from 329 h to 193 h during the study period. Additionally, the telepsychiatry intervention reduced the need for face-to-face evaluations by 75%.

#### Crisis intervention

Crisis intervention was explored in eight studies (27%) focusing on diverse mental health conditions in CYP, such as anxiety, depression and self-harm.^27,31,32,37,38^ The average duration for a crisis intervention was 4 days. Among the eight studies that reported outcomes associated with a crisis intervention, there was only one study that showed a reduction in the LOS and the frequency of emergency department return visits. One high-quality study^40^ reported a 15% decrease in 30-day emergency department return visits among the intervention group. Moreover, a moderate-quality cohort study^27^ demonstrated significant reductions in emergency department LOS following the ‘Child Guidance’ intervention, with a mean decrease of 43.10 min (*P* < 0.001). Additional findings can be found in Table 2.^30,41^

#### General services

General services for CYP mental health included psychiatric evaluations, treatment plan reviews, psychosocial treatments, psychoeducation for families and behavioural health treatments. In the reviewed studies (23%), these services, examined through cohort and pre–post intervention studies, benefited CYP aged 3–17 years with various mental health diagnoses (anxiety, autism spectrum disorder, attention-deficit hyperactivity disorder, conduct disorders, depression, eating disorders, psychotic disorders, self-harm/suicidal ideation) (Table 2).

One study indicated an 8% reduction in emergency department presentations (32.8–24.5%) after receiving intervention services.^28^ Another study focusing on psychosis intervention found significant improvements in CYP behaviour disorders and psychosis, evidenced by improved Children's Global Assessment Scale (CGAS) scores at assessment and discharge.^38^ Interventions, lasting from 3 h to <3 months, demonstrated overall benefits, improving outcomes in behaviour disorders and reducing emergency department presentations.^18,20,26,28,39,45,47^

#### Group therapy

Group therapy, including family therapy, psychotherapy, wellness and substance misuse counselling, demonstrated effectiveness, notably in reducing hospital readmission rates for CYP in family-based interventions.^34^ Older adolescents in these therapies exhibited diverse mental health symptoms (anxiety, autism spectrum, behavioural disorders, depression, eating disorders, psychosis, self-harm/suicidal ideation)^23,25,35,42,45^

Cognitive–behavioural therapy (CBT) was evaluated in four low-level evidence studies (14%)^24,39,45,46^ of CBT programmes for CYP (age range: 8–16 years).^24,44,49,51^ A group-based CBT programme effectively reduced suicidal ideation at 30 days and 3 months post-intervention for adolescents aged 12–17 years.^24^ Additionally, CBT interventions for older adolescents (mean age 16 years) resulted in significant improvements in anxiety and mood, without cases of deterioration.^46^

#### In-hospital treatment

In five studies (17%) centred on in-hospital interventions for CYP aged 8–21 years, two were pre-and post-studies^26,45^ and three were cohort studies,^31,34,38^ with risks of bias ranging from moderate to high. Diagnoses included adjustment disorders, anxiety, autism spectrum disorder, behavioural disorders, depression, eating disorders, psychosis and self-harm/suicidal ideation. In-hospital stays, lasting from 1 to 3 months, tailored interventions to mental health conditions. One study reported multimodal in-hospital interventions for CYP showed positive outcomes, with emergency evaluation interviews and brief therapeutic interventions effectively treating CYP within 24 h of their emergency department presentation. Hospital admission rates decreased significantly after the intervention, and no control group was provided for comparison.^31,36^

#### Individualised therapy

Two studies^27,41^ with a moderate risk of bias (7%) investigated individualised therapy for CYP.^20,34^ In one study,^20^ a collaborative practice model intervention was associated with increased access to psychiatric evaluations (adjusted odds ratio 4.16, *P* < 0.01) and greater engagement in follow-up sessions (adjusted odds ratio 7.54, *P* < 0.01) for CYP with behaviour, anxiety and mood disorders. The other study found that young people with eating disorders who received weekly individual therapy in a partial hospital programme had significantly lower LOS (29.37 days, s.d. = 18.85 days) compared with the control group (32.96 days, s.d. = 14.59 days), along with lower hospital readmission rates (*P* < 0.04).^34^

#### Integrated services

Integrated services, comprising various components such as integration with primary care, linkage to specialty and community mental health services, and paediatric behavioural interventions, were explored in eight studies.^19,21,24,38,45,47^ These services had a duration of <3 months and were accessed by CYP aged 0–17 years with conditions like adjustment disorder,^38^ aggression and anxiety,^47^ autism spectrum disorder,^25,45,52^ behaviour disorders,^38^ depression and mood disorders,^38,45^ eating disorders,^38,45^ psychotic disorders,^38,45^ self-harm/suicidal ideation^24,38,45,47^ and substance misuse.^47^ Two Canadian studies provided moderate-level evidence on out-patient aftercare services, showing mixed results in terms of emergency department readmissions.^43,44^ Another study highlighted the positive impact of a multidisciplinary mobile youth outreach service on consumer engagement and hostility risk in CYP with psychotic disorders.^47^

#### Parent–child dyadic therapy

Parent–child dyadic therapy was the focus of one study with a moderate level of bias and low-quality evidence.^22^ The study evaluated an infant preschool family mental health initiative for young children (mean age 3 years, range 0–5 years) with anxiety, behavioural disorders, adjustment disorder and affective or reactive disorders. The intervention employed relationship-based dyadic techniques with parents and their children, resulting in high parental satisfaction and significant improvements in the Mental Health Screening Tool and Moderate Risk Assessment scores, as well as higher scores on the Parent–Infant Relationship Global Assessment Scale and Global Assessment of Functioning Scale at post-test.^22^

#### Pharmacotherapy

Five studies (16%) evaluated pharmacotherapy efficacy for CYP aged 4–16 years.^18,23,25,39,45^ Medication, combined with other therapies, showed significant improvements in mental health symptoms, particularly for externalising symptoms (Cohen's *d* = 0.99) and total problems (Cohen's *d* = 0.86).^23^ Older adolescents received pharmacotherapy for a range of mental health conditions, such as attention-deficient hyperactivity disorder, autism and depression.^18,25,39^ However, some studies lacked specific medication details (type and dose) and effects.^18,45^ An early psychosis intervention service employing medication management and multimodal therapies, including psychiatric evaluations, psychoeducation (i.e. healthy lifestyle choices, symptom awareness, medication management), individual and family therapy sessions showed improved CGAS scores.^39^ Additional details associated with the outcomes for each study can be found in Table 1, where a summary of the results from each research study are provided.

#### Safety and risk assessment planning

Three studies (10%) examined safety and risk assessment planning services, all with a moderate risk of bias. Safety and risk assessments for younger children (age 2–5 years) primarily focused on those with behavioural disorders,^23^ whereas for adolescents (age 12–17 years), the services targeted individuals with self-harm/suicidal ideation^24^ and behavioural disorders.^30^ In a study with moderate evidence, safety planning was integrated into a care service for young people with self-harm/suicidal ideation,^19^ and outcomes were compared with a control group. Among the participants, 26% received behavioural safety planning as part of the intervention, and the results showed no significant difference in 30- or 90-day emergency department return rates compared with the control group.^19^

## Discussion

The aim of this study was to investigate whether brief interventions, incorporating established screening procedures and delivered in out-patient settings (excluding emergency departments), effectively decreased the reliance on emergency department assessments, re-evaluations or in-patient admissions among CYP experiencing mental health crises. This systematic review included studies exploring a wide range of brief interventions in different settings for CYP presenting with a mental health crisis. To translate these findings into practical strategies, several key considerations need to be considered.

In the context of the emergency department, the use of measurement-based care (MBC) for screening purposes can help identify CYP who require brief interventions and improve their treatment outcomes.^52,54^ MBC can be used by trained health professionals to track treatment progress and outcomes, and has been shown to improve treatment outcomes for CYP with mental health conditions. A study by Parikh et al^55^ found that the use of standardised screening tools in the emergency department improved the identification of mental health disorders and increased the provision of appropriate referrals and interventions. Another study by Chun et al^56^ found that the use of screening tools in the emergency department improved the identification of mental health conditions among CYP. Further, Bickman et al^57^ found that the use of MBC led to significant improvements in mental health outcomes for youths in community mental health clinics, including a reduction in symptoms and improvement in overall functioning.

Integrated services are core strategies to be employed in brief interventions in mental health programmes. Drake et al^58^ found that the use of a care coordination model was a key component of successful implementation of evidence-based practices in routine mental health service settings. The authors emphasised the importance of considering regional disparities between urban and rural healthcare systems in the implementation of programmes. A study by Probst et al^59^ found that rural areas face unique challenges in implementing integrated service programmes, because of limited resources and access to care. Hoffman et al^60^ examined the association between follow-up care visits and return mental health acute care encounters among CYP who had received mental health emergency care, and showed that CYP who received follow-up care visits within 7 and 30 days after an initial mental health emergency visit were less likely to have return mental health acute care encounters. These findings suggest that follow-up care is crucial in reducing the risk of subsequent acute care encounters among CYP with mental health conditions. Further, Lyon and Bruns^61^ also found that factors such as a reduction in symptom severity and family engagement were associated with the likelihood of follow-up care after brief interventions for CYP with behavioural health needs.

Crisis interventions also play key role in emergency department LOS. For example, the ‘Child Guidance’ intervention contributed to significant reductions in emergency department LOS. The Child Guidance intervention is a collaborative model that involves a full-time psychiatric social worker and a full-time child psychiatrist.^24^ It is specifically designed to provide efficient mental healthcare to children with volatile mood disorders in the emergency department. This innovative approach ensures that CYP with acute mental health needs receive timely and specialised care, resulting in notable reductions in emergency department LOS. The success of the Child Guidance intervention highlights its potential as an effective and efficient strategy for optimising the care and outcomes of CYP in crisis situations.^24^

The implementation of brief interventions may require additional funding and resources. Therefore, it is important to consider the cost-effectiveness surrounding them when developing and implementing these programmes. For example, a study by Grist et al^62^ found that a brief intervention for CYP with anxiety disorders was cost-effective compared with usual care.^62,63^

Screening CYP to determine the appropriate intervention can be challenging. However, validated screening tools are available to identify CYP who require brief interventions. For example, the Pediatric Symptom Checklist is a widely used screening tool to identify CYP with mental health conditions in primary care settings,^64^ whereas the ‘Home, Education, Activities/peers, Drugs/alcohol, Suicidality, Emotions/behavior, Discharge resources' tool has been shown to be effective in identifying CYP who require emergency department-based interventions.^65^ Moreover, the Suicide Assessment Five-Step Evaluation and Triage is also a widely used tool for assessing suicide risk in individuals, including CYP who present with suicidal ideation.^66^ Additionally screening tools, such as the CGAS^67^ and the Screen for Child Anxiety Related Emotional Disorders,^68^ may also be useful in identifying CYP who require brief interventions.^69,70^

Addressing the barriers to follow-up care is important for improving treatment outcomes for CYP who receive brief interventions. Recent studies have highlighted the importance of follow-up care for CYP with mental health conditions. For example, a study by Katon et al^71^ found that regular follow-up care was associated with improved mental health outcomes for CYP with depression. A study by Zima et al^72^ found that many CYP with mental health conditions face barriers in accessing mental health services, such as a lack of available services in their area or difficulty accessing care because of transportation issues. In addition, stigma and shame surrounding mental health issues can also be a barrier to care for some CYP.^73^ Interventions that address these barriers, such as providing culturally sensitive care and enhancing communication between healthcare providers and families, have been shown to improve follow-up rates among CYP with mental health conditions.^74^

Long-term data collection is also an important aspect in evaluating the effectiveness of mental health for CYP, and recent studies have emphasised the significance of conducting follow-up assessments to assess treatment outcomes over extended periods. Weisz et al^75^ observed that CBT demonstrated sustained benefits for anxiety and depression in CYP, evident even at a 5-year follow-up assessment. Moreover, a meta-analysis conducted by Bickman et al^57^ indicated that mental health treatments for CYP generally maintained their effects over time, with potential implications that longer treatment durations could yield more lasting results. Although our systematic review did not explicitly incorporate long-term data supporting the findings, we recognise the importance of considering such data to enhance our understanding of mental health treatment effectiveness. Future research efforts should identify factors contributing to sustained treatment effects and develop interventions promoting enduring mental health outcomes for CYP.

### Limitations

Given the escalating number of CYP who present to the emergency department in crisis, it is plausible that brief interventions may enhance mental health outcomes for this population. Consequently, it is imperative to systematically assess the outcomes of brief interventions against a comparison group (including pre-intervention controls), utilising consistent measurement tools to investigate their effectiveness in lowering emergency department presentations, in-patient admissions, LOS, as well as the psychological impact on CYP and their families. Further research is needed to identify impediments to the effective implementation of these interventions, as well as high-quality studies that can compare different interventions in terms of consumer outcomes and perspectives, with appropriate control groups. Moreover, it is crucial to assess the impact of offering brief interventions on other parts of the mental health treatment services sector, such as private services, primary healthcare and community-based public mental health services.

In conclusion, this systematic review examined the impact of brief interventions, incorporating established screening procedures and delivered in out-patient settings, on the utilisation of emergency department assessments, re-evaluations and in-patient admissions among CYP experiencing mental health crises. Findings provide useful insights to guide and support the development of new and existing brief interventions for consumers with mental health concerns and their families/caregivers. The findings indicate that brief interventions can be successfully delivered in various out-patient settings, such as linking clients to community or out-patient services or in-home care, leading to a reduction in hospital readmission rates and LOS in hospital. This review provided moderate evidence to indicate that incorporating family-based therapies into hospital programmes improves mental health outcomes for CYP in the short term, whereas other lower-quality evidence supports multimodal treatments, including parent–child dyadic therapy and CBT.

However, the feasibility and acceptability of lower-quality evidenced brief interventions for CYP and their families/caregivers requires further research, with a pre-intervention comparison group, in assessing their effectiveness in reducing symptoms and improving mental health function and quality of life across a wide spectrum of mental health symptoms, severity and age groups. As a rationale for improving mental health outcomes for CYP, brief interventions should consider patient safety, care integration and quality of care, as well as rigorous and consistent evaluation of new brief interventions and therapies. Finally, given that these interventions were typically delivered over a short period (often 8 weeks), long-term follow-up is necessary to determine their sustained effectiveness and success.

## Supporting information

Eapen et al. supplementary material 1 Eapen et al. supplementary material

Eapen et al. supplementary material 2 Eapen et al. supplementary material

## Data Availability

Data availability is not applicable to this article as no new data were created or analysed in this study.

## References

[1] Young Minds Matter. *Adolescent Mental Health. Prevalence of Mental Disorders in Australian Children and Adolescents*. Telethon Kids Institute, 2023 (https://youngmindsmatter.telethonkids.org.au/our-research/prevalence-of-mental-disorders/).

[2] Sara G, Wu J, Uesi J, Jong N, Perkes I, Knight K, et al. Growth in emergency department self-harm or suicidal ideation presentations in young people: comparing trends before and since the COVID-19 first wave in New South Wales, Australia. Aust N Z J Psychiatry 2023; 57(1): 58–68. 35266405 10.1177/00048674221082518 PMC9791324

[3] Kim-Cohen J, Caspi A, Moffitt TE, Harrington H, Milne BJ, Poulton R. Prior juvenile diagnoses in adults with mental disorder: developmental follow-back of a prospective-longitudinal cohort. Arch Gen Psychiatry 2003; 60(7): 709–17. 12860775 10.1001/archpsyc.60.7.709

[4] Wasserman D, Cheng QI, Jiang G-X. Global suicide rates among young people aged 15–19. World Psychiatry 2005; 4(2): 114. 16633527 PMC1414751

[5] Gromada A, Rees G. *Worlds of Influence: Understanding What Shapes Child Well-being in Rich Countries*. United Nations International Children's Emergency Fund, 2020 (https://www.unicef-irc.org/publications/pdf/RCWP-Report-2020-WoI.pdf).

[6] Bould H, Mars B, Moran P, Biddle L, Gunnell D. Rising suicide rates among adolescents in England and Wales. Lancet 2019; 394(10193): 116–7. 31227370 10.1016/S0140-6736(19)31102-X

[7] Skinner R, McFaull S, Draca J, Frechette M, Kaur J, Pearson C, et al. Suicide and self-inflicted injury hospitalizations in Canada (1979 to 2014/15). Health Promot Chronic Dis Prev Can 2016; 36(11): 243–51. 27882859 10.24095/hpcdp.36.11.02 PMC5432047

[8] Hu N, Nassar N, Shrapnel J, Perkes I, Hodgins M, O'Leary F, et al. The impact of the COVID-19 pandemic on paediatric health service use within one year after the first pandemic outbreak in New South Wales Australia – a time series analysis. Lancet Reg Health West Pac 2022; 19: 100311. 34746898 10.1016/j.lanwpc.2021.100311 PMC8564784

[9] John J, Synn EP, Winata T, Eapen V, Lin P-I. Increased ambulance attendances related to suicide and self-injury in response to the pandemic in Australia. Aust N Z J Psychiatry 2023; 57(1): 140–2. 36062782 10.1177/00048674221121090 PMC10076165

[10] Deighton J, Croudace T, Fonagy P, Brown J, Patalay P, Wolpert M, et al. Measuring mental health and wellbeing outcomes for children and adolescents to inform practice and policy: a review of child self-report measures. Child Adolesc Psychiatry Ment Health 2014; 8: 14. 24834111 10.1186/1753-2000-8-14 PMC4022575

[11] Eapen V, Stylianakis A, Scott E, Milroy H, Bowden M, Haslam R, et al. Stemming the tide of mental health problems in young people: challenges and potential solutions. Aust N Z J Psychiatry 2023; 57(4): 482–8. 36377648 10.1177/00048674221136037

[12] Otis M, Barber S, Amet M, Nicholls D. Models of integrated care for young people experiencing medical emergencies related to mental illness: a realist systematic review. Eur Child Adolesc Psychiatry 2023; 32(12): 2439–52. 36151355 10.1007/s00787-022-02085-5 PMC9510153

[13] Clisu DA, Layther I, Dover D, Viner RM, Read T, Cheesman D, et al. Alternatives to mental health admissions for children and adolescents experiencing mental health crises: a systematic review of the literature. Clin Child Psychol Psychiatry 2022; 27(1): 35–60. 34836461 10.1177/13591045211044743 PMC8811329

[14] Newton AS, Hartling L, Soleimani A, Kirkland S, Dyson MP, Cappelli M. A systematic review of management strategies for children's mental health care in the emergency department: update on evidence and recommendations for clinical practice and research. Emerg Med J 2017; 34(6): 376–84. 28119350 10.1136/emermed-2016-205939

[15] Page MJ, McKenzie JE, Bossuyt PM, Boutron I, Hoffmann TC, Mulrow CD, et al. The PRISMA 2020 statement: an updated guideline for reporting systematic reviews. Int J Surg 2021; 88: 105906. 33789826 10.1016/j.ijsu.2021.105906

[16] Ouzzani M, Hammady H, Fedorowicz Z, Elmagarmid A. Rayyan – a web and mobile app for systematic reviews. Syst Rev 2016; 5(1): 210. 27919275 10.1186/s13643-016-0384-4 PMC5139140

[17] Popay J, Roberts H, Sowden A, Petticrew M, Arai L, Rodgers M, et al. Guidance on the Conduct of Narrative Synthesis in Systematic Reviews. A Product from the ESRC Methods Programme Version. ESRC Methods Programme, 2006 (https://www.lancaster.ac.uk/media/lancaster-university/content-assets/documents/fhm/dhr/chir/NSsynthesisguidanceVersion1-April2006.pdf).

[18] Aupont O, Doerfler L, Connor DF, Stille C, Tisminetzky M, McLaughlin TJ. A collaborative care model to improve access to pediatric mental health services. Adm Policy Ment Health Ment Health Serv Res 2013; 40(4): 264–73. 10.1007/s10488-012-0413-0 22527709

[19] Casher GA, Sutton B, Roosevelt G, Simpson SA. Evaluation of an integrated psychology service in a pediatric emergency department and urgent care. Pediatr Emerg Care 2022; 38: E697–702. 34137565 10.1097/PEC.0000000000002328

[20] Grimes KE, Creedon TB, Webster CR, Coffey SM, Hagan GN, Chow CM. Enhanced child psychiatry access and engagement via integrated care: a collaborative practice model with pediatrics. Psychiatr Serv 2018; 69(9): 986–92. 30041586 10.1176/appi.ps.201600228

[21] Holder SM, Rogers K, Peterson E, Shoenleben R, Blackhurst D. The impact of mental health services in a pediatric emergency department the implications of having trained psychiatric professionals. Pediatr Emerg Care 2017; 33: 311–4. 27668915 10.1097/PEC.0000000000000836

[22] Knapp PK, Ammen S, Arstein-Kerslake C, Poulsen MK, Mastergeorge A. Feasibility of expanding services for very young children in the public mental health setting. J Am Acad Child Adolesc Psychiatry 2007; 46: 152–61. 17242618 10.1097/01.chi.0000246058.68544.35

[23] Martin SE, McConville DW, Williamson LR, Feldman G, Boekamp JR. Partial hospitalization treatment for preschoolers with severe behavior problems: child age and maternal functioning as predictors of outcome. Child Adolesc Ment Health 2013; 18: 24–32. 32847260 10.1111/j.1475-3588.2012.00661.x

[24] McBee-Strayer SM, Thomas GV, Bruns EM, Heck KM, Alexy ER, Bridge JA. Innovations in practice: intensive crisis intervention for adolescent suicidal ideation and behavior – an open trial. Child Adolesc Ment Health 2019; 24: 345–9. 32677346 10.1111/camh.12340

[25] Cummings MR, Dubovsky SL, Ehrlich I, Kandefer S, Van Cleve J, Yin Y, et al. Preliminary assessment of a novel continuum-of-care model for young people with autism spectrum disorders. Psychiatr Serv 2020; 71(12): 1313–6. 32988326 10.1176/appi.ps.201900574

[26] Hasken C, Wagers B, Sondhi J, Miller J, Kanis J. The impact of a new on-site inpatient psychiatric unit in an urban pediatric emergency department. Pediatr Emerg Care 2022; 38(1): e12–e6. 32658116 10.1097/PEC.0000000000002177

[27] Mahajan P, Thomas R, Rosenberg DR, Leleszi JP, Leleszi E, Mathur A, et al. Evaluation of a child guidance model for visits for mental disorders to an inner-city pediatric emergency department. Pediatr Emerg Care 2007; 23(4): 212–7. 17438432 10.1097/PEC.0b013e31803e177f

[28] Parast L, Bardach NS, Burkhart Q, Richardson LP, Murphy JM, Gidengil CA, et al. Development of new quality measures for hospital-based care of suicidal youth. Acad Pediatr 2018; 18(3): 248–55. 29100860 10.1016/j.acap.2017.09.017 PMC12167927

[29] Reliford A, Adebanjo B. Use of telepsychiatry in pediatric emergency room to decrease length of stay for psychiatric patients, improve resident on-call burden, and reduce factors related to physician burnout. Telemed e-Health 2019; 25(9): 828–32. 10.1089/tmj.2018.0124 30379635

[30] Sheridan DC, Sheridan J, Johnson KP, Laurie A, Knapper A, Fu R, et al. The effect of a dedicated psychiatric team to pediatric emergency mental health care. J Emerg Med 2016; 50(3): e121–e8. 26803193 10.1016/j.jemermed.2015.10.034

[31] Uspal NG, Rutman LE, Kodish I, Moore A, Migita RT. Use of a dedicated, non–physician-led mental health team to reduce pediatric emergency department lengths of stay. Acad Emerg Med 2016; 23(4): 440–7. 26806468 10.1111/acem.12908

[32] Rogers SC, Griffin LC, Masso Jr PD, Stevens M, Mangini L, Smith SR. CARES: improving the care and disposition of psychiatric patients in the pediatric emergency department. Pediatr Emerg Care 2015; 31(3): 173–7. 25706924 10.1097/PEC.0000000000000378

[33] Kells M, Schubert-Bob P, Nagle K, Hitchko L, O'Neil K, Forbes P, et al. Meal supervision during medical hospitalization for eating disorders. Clin Nurs Res 2017; 26(4): 525–37. 26964805 10.1177/1054773816637598

[34] Huryk KM, Casasnovas AF, Feehan M, Paseka K, Gazzola P, Loeb KL. Lower rates of readmission following integration of family-based treatment in a higher level of care. Eating Disorders 2021; 29(6): 677–84. 33135596 10.1080/10640266.2020.1823173

[35] McDowell G, Valleru J, Adams M, Fristad MA. Centering, affective regulation, and exposure (CARE) group: mindful meditation and movement for youth with anxiety. Evid Based Pract Child Adolesc Ment Health 2020; 5(2): 139–46.

[36] Gillig PM. Child & adolescent psychiatry: an adolescent crisis service in a rural area. Psychiatr Serv 2004; 55(12): 1363–5. 15574408 10.1176/appi.ps.55.12.1363

[37] Wharff EA, Ginnis KM, Ross AM. Family-based crisis intervention with suicidal adolescents in the emergency room: a pilot study. Soc Work 2012; 57(2): 133–43. 23038875 10.1093/sw/sws017

[38] Greenham SL, Bisnaire L. An outcome evaluation of an inpatient crisis stabilization and assessment program for youth. Resident Treat Child Youth 2008; 25(2): 123–43.

[39] Morris A, Nixon MK, Keyes R, Ashmore D. Early psychosis intervention service for children and youth: a retrospective chart review of the first four years. Early Interv Psychiatry 2009; 3(2): 99–107. 21352183 10.1111/j.1751-7893.2009.00115.x

[40] Ishikawa T, Chin B, Meckler G, Hay C, Doan Q. Reducing length of stay and return visits for emergency department pediatric mental health presentations. Can J Emerg Med 2021; 23(1): 103–10. 10.1007/s43678-020-00005-7 33683603

[41] Parker KCH, Roberts N, Williams C, Benjamin M, Cripps L, Woogh C. Urgent adolescent psychiatric consultation: from the accident and emergency department to inpatient adolescent psychiatry. J Adolesc 2003; 26(3): 283–93. 12770527 10.1016/s0140-1971(03)00014-9

[42] Gusella JL, Campbell AG, Lalji K. A shift to placing parents in charge: does it improve weight gain in youth with anorexia? Paediatr Child Health 2017; 22(5): 269–72. 29479232 10.1093/pch/pxx063 PMC5804961

[43] Cheng C, Chan CWT, Gula CA, Parker MD. Effects of outpatient aftercare on psychiatric rehospitalization among children and emerging adults in Alberta, Canada. Psychiatr Serv 2017; 68(7): 696–703. 28245702 10.1176/appi.ps.201600211

[44] Carlisle CE, Mamdani M, Schachar R, To T. Aftercare, emergency department visits, and readmission in adolescents. J Am Acad Child Adolesc Psychiatry 2012; 51(3): 283–93.e4. 22365464 10.1016/j.jaac.2011.12.003

[45] Adrian N, Smith JG. Occupied bed days a redundant currency? An evaluation of the first 10 years of an integrated model of care for mentally ill adolescents. Clin Child Psychol Psychiatry 2015; 20(3): 458–71. 24694901 10.1177/1359104514527298

[46] Sclare I, Michelson D, Malpass L, Coster F, Brown J. Innovations in practice: dISCOVER CBT workshops for 16–18-year-olds: development of an open-access intervention for anxiety and depression in inner-city youth. Child Adolesc Ment Health 2015; 20: 102–6. 32680391 10.1111/camh.12060

[47] Schley C, Yuen K, Fletcher K, Radovini AJ. Does engagement with an intensive outreach service predict better treatment outcomes in ‘high-risk’ youth? Early Interv Psychiatry 2012; 6(2): 176–84. 22273358 10.1111/j.1751-7893.2011.00338.x

[48] Chen A, Dinyarian C, Inglis F, Chiasson C, Cleverley K. Discharge interventions from inpatient child and adolescent mental health care: a scoping review. Eur Child Adolesc Psychiatry 2022; 31(6): 857–78. 32886222 10.1007/s00787-020-01634-0 PMC9209379

[49] Asarnow JR, Jaycox LH, Tang L, Duan N, LaBorde AP, Zeledon LR, et al. Long-term benefits of short-term quality improvement interventions for depressed youths in primary care. Am J Psychiatry 2009; 166: 1002–10. 19651711 10.1176/appi.ajp.2009.08121909

[50] Richardson LP, Ludman E, McCauley E, Lindenbaum J, Larison C, Zhou C, et al. Collaborative care for adolescents with depression in primary care: a randomized clinical trial. JAMA 2014; 312(8): 809–16. 25157724 10.1001/jama.2014.9259 PMC4492537

[51] Rickwood DJ, Mazzer KR, Telford NR, Parker AG, Tanti CJ, McGorry PD. Changes in psychological distress and psychosocial functioning in young people visiting headspace centres for mental health problems. Med J Austr 2015; 202(10): 537–42. 10.5694/mja14.01696 26021366

[52] Jensen-Doss A, Hawley KM. Understanding barriers to evidence-based assessment: clinician attitudes toward standardized assessment tools. J Clin Child Adolesc Psychol 2010; 39(6): 885–96. 21058134 10.1080/15374416.2010.517169 PMC3058768

[53] Garland AF, Brookman-Frazee L, Hurlburt MS, Accurso EC, Zoffness RJ, Haine-Schlagel R, et al. Mental health care for children with disruptive behavior problems: a view inside therapists’ offices. Psychiatr Serv 2010; 61(8): 788–95. 20675837 10.1176/appi.ps.61.8.788 PMC3019612

[54] Chorpita BF, Daleiden EL, Weisz JR. Identifying and selecting the common elements of evidence based interventions: a distillation and matching model. Ment Health Serv Res 2005; 7: 5–20. 15832690 10.1007/s11020-005-1962-6

[55] Parikh A, Fristad MA, Axelson D, Krishna RJ. Evidence base for measurement-based care in child and adolescent psychiatry. Child Adolesc Psychiatr Clin N Am 2020; 29(4): 587–99. 32891364 10.1016/j.chc.2020.06.001

[56] Chun TH, Duffy SJ, Linakis JG. Emergency department screening for adolescent mental health disorders: the who, what, when, where, why and how it could and should be done. Clin Pediatr Emerg Med 2013; 14(1): 3–11. 23682241 10.1016/j.cpem.2013.01.003 PMC3652490

[57] Bickman L, Kelley SD, Breda C, de Andrade AR, Riemer M. Effects of routine feedback to clinicians on mental health outcomes of youths: results of a randomized trial. Psychiatr Serv 2011; 62(12): 1423–9. 22193788 10.1176/appi.ps.002052011

[58] Drake RE, Goldman HH, Leff HS, Lehman AF, Dixon L, Mueser KT, et al. Implementing evidence-based practices in routine mental health service settings. Psychiatr Serv 2001; 52(2): 179–82. 11157115 10.1176/appi.ps.52.2.179

[59] Probst JC, Laditka SB, Wang J-Y, Johnson AO. Effects of residence and race on burden of travel for care: cross sectional analysis of the 2001 US national household travel survey. BMC Health Serv Res 2007; 7: 40. 17349050 10.1186/1472-6963-7-40 PMC1851736

[60] Hoffmann JA, Krass P, Rodean J, Bardach NS, Cafferty R, Coker TR, et al. Follow-up after pediatric mental health emergency visits. Pediatrics 2023; 151(3): e2022057383. 36775807 10.1542/peds.2022-057383 PMC10187982

[61] Lyon AR, Bruns EJ. From evidence to impact: joining our best school mental health practices with our best implementation strategies. School Ment Health 2019; 11: 106–14. 31709018 10.1007/s12310-018-09306-w PMC6839825

[62] Grist R, Croker A, Denne M, Stallard P. Technology delivered interventions for depression and anxiety in children and adolescents: a systematic review and meta-analysis. Clin Child Fam Psychol Rev 2019; 22: 147–71. 30229343 10.1007/s10567-018-0271-8 PMC6479049

[63] Morrissey-Kane E, Prinz RJ. Engagement in child and adolescent treatment: the role of parental cognitions and attributions. Clin Child Fam Psychol Rev 1999; 2: 183–98. 11227074 10.1023/a:1021807106455

[64] Jellinek MS, Murphy JM, Little M, Pagano ME, Comer DM, Kelleher KJ, et al. Use of the pediatric symptom checklist to screen for psychosocial problems in pediatric primary care: a national feasibility study. Arch Pediatr Adolesc Med 1999; 153(3): 254–60. 10086402 10.1001/archpedi.153.3.254 PMC3905751

[65] Cappelli M, Gray C, Zemek R, Cloutier P, Kennedy A, Glennie E, et al. The HEADS-ED: a rapid mental health screening tool for pediatric patients in the emergency department. Pediatrics 2012; 130(2): e321–7. 22826567 10.1542/peds.2011-3798

[66] Substance Abuse and Mental Health Services Administration (SAMHSA). *SAFE-T Pocket Card: Suicide Assessment Five-Step Evaluation and Triage (SAFE-T) for Clinicians*. SAMHSA, 2009 (https://www.samhsa.gov/resource/dbhis/safe-t-pocket-card-suicide-assessment-five-step-evaluation-triage-safe-t-clinicians).

[67] Shaffer D, Gould MS, Brasic J, Ambrosini P, Fisher P, Bird H, et al. A Children's Global Assessment Scale (CGAS). Arch Gen Psychiatry 1983; 40(11): 1228–31. 6639293 10.1001/archpsyc.1983.01790100074010

[68] Birmaher B, Khetarpal S, Brent D, Cully M, Balach L, Kaufman J, et al. The Screen for Child Anxiety Related Emotional Disorders (SCARED): scale construction and psychometric characteristics. J Am Acad Child Adolesc Psychiatry 1997; 36(4): 545–53. 9100430 10.1097/00004583-199704000-00018

[69] Ringeisen H, Henderson K, Hoagwood KJ. Context matters: schools and the ‘research to practice gap’ in children's mental health. School Psychol Rev 2003; 32(2): 153–68.

[70] Birmaher B, Brent DA, Chiappetta L, Bridge J, Monga S, Baugher M, et al. Psychometric properties of the Screen for Child Anxiety Related Emotional Disorders (SCARED): a replication study. J Am Acad Child Psychiatry 1999; 38(10): 1230–6. 10.1097/00004583-199910000-00011 10517055

[71] Katon W, Richardson L, Russo J, McCarty CA, Rockhill C, McCauley E, et al. Depressive symptoms in adolescence: the association with multiple health risk behaviors. Gen Hosp Psychiatry 2010; 32(3): 233–9. 20430225 10.1016/j.genhosppsych.2010.01.008 PMC3671856

[72] Zima BT, Murphy JM, Scholle SH, Hoagwood KE, Sachdeva RC, Mangione-Smith R, et al. National quality measures for child mental health care: background, progress, and next steps. Pediatrics 2013; 131(suppl 1): S38–S49. 23457148 10.1542/peds.2012-1427e PMC4046520

[73] Purtle J, Peters R, Brownson RC. A review of policy dissemination and implementation research funded by the National Institutes of Health, 2007–2014. Implement Sci 2015; 11: 1. 10.1186/s13012-015-0367-1 PMC4700744 26727969

[74] Betancourt JR, Green AR, Carrillo JE, Ananeh-Firempong O. Defining cultural competence: a practical framework for addressing racial/ethnic disparities in health and health care. Public Health Rep 2003; 118(4): 293–302. 12815076 10.1016/S0033-3549(04)50253-4 PMC1497553

[75] Weisz JR, Kuppens S, Ng MY, Eckshtain D, Ugueto AM, Vaughn-Coaxum R, et al. What five decades of research tells US about the effects of youth psychological therapy: a multilevel meta-analysis and implications for science and practice. Am Psychol 2017; 72(2): 79–117. 28221063 10.1037/a0040360

