# Supplementary material for: Evidence-based brief interventions targeting acute mental health presentations for children and adolescents: systematic review
Source: BJPsych Open. 2024 Apr 11;10(3):e78. doi: 10.1192/bjo.2024.25 (PMC11060074; doi:10.1192/bjo.2024.25)
Supplement: Eapen et al. supplementary material 1 — Eapen et al. supplementary material [file S2056472424000255sup001.docx]

**Appendix 1: Quality assessment and data extraction**

Two reviewers (BG, BOA) blindly assessed the quality of extracted studies using the National Health and Medical Research Council (NHMRC) levels of evidence and grade guidelines tool (1). The NHMRC levels of evidence uses four levels of quality that constitute the Evidence Hierarchy (‘levels of evidence’ that are derived according to the type of research question within each study), with levels I (systematic reviews of randomised controlled trials), II (randomised controlled trials) and III-I (pseudorandomised controlled trials) graded as ‘higher’ level scores, elucidating greater confidence associated with the findings of the study. Further, level III-2 (comparative studies with controls such as non-randomised experimental trials, cohort studies, case-control studies, or interrupted time series with a control group) were graded as ‘Moderate’ level of evidence. Levels III-3 (comparative studies without controls such as historical control study, two or more single arm study, interrupted time series without a parallel control group), and IV (case series studies with post-test or pre and post-tests) were indicative of ‘lower levels’ of evidence. Discrepancies were resolved through discussion with a moderator (TW) to reach a consensus.

**Appendix 2: Risk of Bias**

The Methodological Index for Non-randomised Studies (MINORS) score, with a global ideal score of 17 for non-comparative studies (15, 18-24, 26-28, 32, 33, 36, 38, 43, 44) and 13 for comparative studies (16, 17, 25, 29, 30, 31, 34, 35, 37, 39, 40-42) were used to assess risk of bias. Two reviewers (BOA, BG) independently assessed the risk of bias for each of the included studies and scores that could not reach an agreement were moderated by a third reviewer (TN). Following an assessment of each of the thirty studies using the MINORS tool, twelve studies were assessed as having a high risk of bias (18, 23, 24, 26, 28, 29, 32, 33, 36, 38, 44, 46), eighteen as having a moderate risk of bias (15-17, 20, 21, 24, 25, 27, 30, 31, 34, 35, 37, 39, 40-43). There were no included studies that were assessed as having low risk of bias.
