# Supplementary material for: Evidence-based brief interventions targeting acute mental health presentations for children and adolescents: systematic review
Source: BJPsych Open. 2024 Apr 11;10(3):e78. doi: 10.1192/bjo.2024.25 (PMC11060074; doi:10.1192/bjo.2024.25)
Supplement: Eapen et al. supplementary material 2 — Eapen et al. supplementary material [file S2056472424000255sup002.docx]

**Supplementary 1: Search Strategy**

| **Concept** | **Database** | | | | | | |  |
| --- | --- | --- | --- | --- | --- | --- | --- | --- |
|  | **Embase Classic + Embase**  **<2000 to 2022>** | | **Ovid Psychinfo**  **<2000-2022>** | | **PUBMED**  **<2000 to 2022>** | | **Web of Science**  **<2000-2022>** | **Cochrane**  **<2000-2022>** |
|  | **Free text terms** | **Mesh terms** | **Free text terms** | **Mesh terms** | **Free text terms** | **Mesh terms** | **Free text terms** | **Free text terms** |
| Young people aged 0-17 years | child*.mp.  adolesc*.mp.  youth*.mp.  p?ediatric*.mp.  teen*.mp.  pubert*.mp.  young.mp. | adolescent/ or child/  pediatrics/ or pediatric emergency medicine/  pediatrics/ or pediatric crisis  pediatrics/ or pediatric crisis intervention  young people.mp.  young person.mp.  exp Child Psychiatry/ exp Child Psychology  exp Adolescent Psychiatry/ or exp Adolescent Psychotherapy/ or exp Adolescent Psychology/  pediatrics/ or chronically ill children/ | child*.mp.  adolesc*.mp.  youth*.mp.  p?ediatric*.mp.  teen*.mp. | adolescent/ or child/  pediatrics/ or pediatric emergency medicine/  pediatrics/ or pediatric crisis  pediatrics/ or pediatric crisis intervention  young people.mp.  exp Child Psychiatry/ or exp Child Psychotherapy/ or exp Child Psychology/  exp Adolescent Psychiatry/ or exp Adolescent Psychotherapy/ or exp Adolescent Psychology/  pediatrics/ or chronically ill children/ | child*.mp.  adolesc*.mp.  youth*.mp.  p?ediatric*.mp.  teen*.mp.  young people.mp. | adolescent/ or child/  pediatrics/or pediatric emergency medicine/  pediatrics/ or pediatric crisis  pediatrics/ or pediatric crisis intervention | child*  adolesc*  youth*  paediatric*  teen*  young people  young person  “Child Psychology”  “Child Psychotherapy”  “Adolescent Psychiatry”  “Adolescent Psychotherapy”  “Adolescent Psychology”  “chronically ill child*” | child*  adolesc*  youth*  paediatric*  teen*  pubert*  young people  young person  “Child Psychology”  “Child Psychotherapy”  “Adolescent Psychiatry”  “Adolescent Psychotherapy”  “Adolescent Psychology”  “chronically ill child*” |
| Mental Illness | (mental adj3 problem*).mp.  mental health*    mental*  (mental adj3 cris?s).mp.  psychiatr*,mp.  mental illness*.mp.  somat*.mp.  malinger*.mp.  autis*.mp.  eating disorder*.mp.  rumination disorder*.mp.  abnormal behavio?r*.mp.  unusual behavio?r*.mp.  “substance use disorder”/  unexplained behavio?r*.mp.  unexplained symptom*.mp.  self?harm.mp.  suicid*.mp.  substance ?use.mp.  alcohol*.mp.  cannab*.mp.  overdose*.mp.  mental disorder*.mp. | anorexia.mp.  bulimia.mp.  purging.mp.  restrictive food intake.mp.  pica.mp.  drug abuse.mp.  alcohol abuse.mp.  psychosomatic.mp.  Substance-Related Disorders/  mental disorders/ or neurotic disorders/ or obsessive-compulsive disorder/ or panic disorder/ or "bipolar and related disorders"/ or "disruptive, impulse control, and conduct disorders"/ or dissociative disorders/ or "feeding and eating disorders"/ or amnesia/ or cognition disorders/ or "attention deficit and disruptive behavior disorders"/ or child behavior disorders/ or child development disorders, pervasive/ or schizophrenia, childhood/ or personality disorders/ or "schizophrenia spectrum and other psychotic disorders"/ or somatoform disorders/ or substance-related disorders/ or "trauma and stressor related disorders"/  exp Mental Disorders/  mental disease/  mental disease.mp. or exp mental disease/  attempted suicide/  exp Eating Disorders/  exp Somatoform Pain Disorder/ or exp Somatoform Disorders/ | (mental adj3 problem*).mp.  (mental adj3 cris?s).mp.  psychiatr*.mp.  mental illness*.mp.  somat*.mp.  malinger*.mp.  autis*.mp.  eating disorder*.mp.  rumination disorder*.mp.  abnormal behavio?r*.mp.  unusual behavio?r*.mp.  unexplained behavio?r*.mp.  unexplained symptom*.mp.  self?harm.mp.  suicid*.mp.  substance ?use.mp.  alcohol*.mp.  cannab*.mp.  overdose*.mp.  mental disorder*.mp.  "substance use disorder"/ | mental disease.mp. or exp mental disease/  psychosomatic.mp.  anorexia.mp.  bulimia.mp.  purging.mp.  restrictive food intake.mp.  pica.mp.  drug abuse.mp.  Substance-Related Disorders/  mental disorders/ or neurotic disorders/ or obsessive-compulsive disorder/ or panic disorder/ or "bipolar and related disorders"/ or "disruptive, impulse control, and conduct disorders"/ or dissociative disorders/ or "feeding and eating disorders"/ or amnesia/ or cognition disorders/ or "attention deficit and disruptive behavior disorders"/ or child behavior disorders/ or child development disorders, pervasive/ or schizophrenia, childhood/ or personality disorders/ or "schizophrenia spectrum and other psychotic disorders"/ or somatoform disorders/ or substance-related disorders/ or "trauma and stressor related disorders"/  exp Mental Disorders/  attempted suicide/  exp Eating Disorders/  exp Somatoform Pain Disorder/ or exp Somatoform Disorders/  mental disease/ | (mental adj3 problem*).mp.  (mental adj3 cris?s).mp.  psychiatr*.mp.  mental illness*.mp.  somat*.mp.  psychosomatic.mp.  malinger*.mp.  autis*.mp.  binge?eating.mp.  rumination disorder*.mp.  abnormal behavio?r*.mp.  unusual behavio?r*.mp.  unexplained behavio?r*.mp.  unexplained symptom*.mp.  self?harm.mp.  suicid*.mp.  substance ?use.mp.  alcohol*.mp.  cannab*.mp.  overdose*.mp. | anorexia.mp.  bulimia.mp.  purging.mp.  restrictive food intake.mp.  pica.mp.  drug abuse.mp  Substance-Related Disorders/  mental disorders/ or “bipolar and related disorders” /or “disruptive, impulse control, and conduct disorders”/ or dissociative disorders/ or “feeding and eating disorders”/ or amnesia/ or cognition disorders/ or “attention deficit and disruptive behaviour disorders”/ or child behaviour disorders/ or child development disorders, pervasive/ or schizophrenia, childhood/ or neurotic disorders/ or somatoform disorders/ or substance-related disorders/ or “trauma and stressor related disorders” /or “behavioural disciplines and activities”/ | mental near/2 problem*  mental near/2 cris*  psychiatr*  mental illness*  somat*  “psychosomatic”  alcohol*  alcohol abuse.mp.  malinger*  autis*  anorexia  purging  “eating disorder”  “restrictive food intake”  “rumination disorder”  pica  “abnormal behaviour*”  “unusual behaviour*”  “abnormal behavior*”  “unusual behavior*”  “unexplained behaviour*”  “unexplained behavior*”  “unexplained symptom*”  “self harm”  self-harm  suicid*  “substance use*”  “substance abuse”  alcohol*  cannab*  overdose*  “drug abuse”  “substance-related disorders”  “neurotic disorder*”  “mental disorder*”  “obsessive-compulsive disorder”  “panic disorder”  “bipolar and related disorders”  “disruptive, impulse control and conduct disorders”  “feeding and eating disorders”  amnesia  “cognition disorders”  “attention deficit and disruptive behavior disorders”  “child behavior disorders”  “attention deficit and disruptive behaviour disorders”  “dissociative disorder*”  “child behaviour disorders”  “child development disorders, pervasive”  schizophrenia, childhood  personality disorders  “schizophrenia spectrum and other psychotic disorders”  “somatoform disorder*”  “substance-related disorders”  “trauma and stressor related disorders”  bulimi*  “binge eating”  “behavioural disciplines and activities”  “attempted suicide”  “somatoform pain disorder” | mental near/2 problem*  mental near/2 cris*  psychiatr*  mental illness*  somat*  “psychosomatic”  malinger*  alcohol*  alcohol abuse.mp.  autis*  anorexia  purging  “eating disorder”  “restrictive food intake”  “rumination disorder”  pica  “abnormal behaviour*”  “unusual behaviour*”  “abnormal behavior*”  “unusual behavior*”  “unexplained behaviour*”  “unexplained behavior*”  “unexplained symptom*”  “self harm”  self-harm  suicid*  “substance use*”  “substance abuse”  alcohol*  cannab*  overdose*  “drug abuse”  “substance-related disorders”  “neurotic disorder*”  “mental disorder*”  “obsessive-compulsive disorder”  “panic disorder”  “bipolar and related disorders”  “disruptive, impulse control and conduct disorders”  “feeding and eating disorders”  amnesia  “cognition disorders”  “attention deficit and disruptive behavior disorders”  “child behavior disorders”  “attention deficit and disruptive behaviour disorders”  “dissociative disorder*”  “child behaviour disorders”  “child development disorders, pervasive”  schizophrenia, childhood  personality disorders  “schizophrenia spectrum and other psychotic disorders”  “somatoform disorder*”  “substance-related disorders”  “trauma and stressor related disorders”  bulimi*  “binge eating”  “behavioural disciplines and activities”  “attempted suicide”  “somatoform pain disorder” |
| Intervention | brief intiv*.mp.  crisis interven*.mp.  famil*.mp.  cognitive* behav*.mp.  dialect* behav*.mp.  well*.mp.  counsel*.mp.  family therapy.mp.  individ*consel*.mp.  psycho* therapy*.mp.  parent-child dyad  healthcare* deliv*.mp.  invidi* counsel*.mp  parent*therap*.mp.  school based therp*.mp.  psycho care.mp.  group session*.mp.  group thera*.mp.  counsel*.mp.  care co-ordin*.mp.  home* care.mp.  patient cent* care.mp.  community based care.mp.  holistic care.mp.  safety care.mp.  risk assessmen*.mp.  In hospital*.mp.  drug counsel*.mp.  pharmacotherapy*.mp.  medication manage*.mp.  assessment serv*.mp.  model of care.mp.  delivery of care.mp.  medical home care.mp.  integr* care.mp.  integrated care*  emergency admission*.mp.  school*.mp.  wrap-around care*.mp.  emergency readmission*.mp.  rehospitali*.mp.  (hospital adj3 emergenc*).mp.  (emergenc* adj2 medic*).mp.  acute hospital*.mp.  (acute adj2 medic*).mp.  (emergenc* adj2 treatment*).mp.  re?feeding.mp.  (emergenc* adj2 in?patient).mp.  (non?elective adj2 care).mp.  (non?elective adj2 treatment).mp.  (unscheduled adj2 care).mp.  (unscheduled adj2 treatment).mp.  (unscheduled adj2 medical).mp.  (non?elective adj2 medical).mp.  (unplanned adj2 care).mp.  (unplanned adj2 treatment).mp.  (unplanned adj2 medical).mp.  (prospective adj2 care).mp.  (prospective adj2 treatment).mp.  (prospective adj2 medical).mp.  (urgent adj2 care).mp.  (urgent adj2 treatment).mp.  (urgent adj2 medical).mp.  emergency hospitali?ation*.mp.  acute hospitali?ation.mp.  acute treatment*.mp.  acute admission*.mp. | brief intiv*.mp.  crisis interven*.mp.  famil*.mp.  cognitive* behav*.mp.  dialect* behav*.mp.  well*.mp.  counsel*.mp.  family therapy.mp.  individ*consel*.mp.  psycho* therapy*.mp.  parent-child dyad  healthcare* deliv*.mp.  invidi* counsel*.mp  parent*therap*.mp.  school based therp*.mp.  psycho care.mp.  group session*.mp.  group thera*.mp.  counsel*.mp.  care co-ordin*.mp.  home* care.mp.  patient cent* care.mp.  community based care.mp.  holistic care.mp.  safety care.mp.  risk assessmen*.mp.  In hospital*.mp.  drug counsel*.mp.  pharmacotherapy*.mp.  medication manage*.mp.  assessment serv*.mp.  model of care.mp.  delivery of care.mp.  medical home care.mp.  integr* care.mp.  integrated care*  emergency admission*.mp.  school*.mp.  wrap-around care*.mp.  emergency medicine/ or pediatric emergency medicine/  emergency medicine/  emergency treatment/  acute care.mp.  acute ward.mp.  emergency services/ or crisis intervention services/ or emergency medicine/  hospital emergency service/ or emergency health service/ | brief intiv*.mp.  crisis interven*.mp.  famil*.mp.  cognitive* behav*.mp.  dialect* behav*.mp.  well*.mp.  counsel*.mp.  family therapy.mp.  individ*consel*.mp.  psycho* therapy*.mp.  parent-child dyad  healthcare* deliv*.mp.  invidi* counsel*.mp  parent*therap*.mp.  school based therp*.mp.  psycho care.mp.  group session*.mp.  group thera*.mp.  counsel*.mp.  care co-ordin*.mp.  home* care.mp.  patient cent* care.mp.  community based care.mp.  holistic care.mp.  safety care.mp.  risk assessmen*.mp.  In hospital*.mp.  drug counsel*.mp.  pharmacotherapy*.mp.  medication manage*.mp.  assessment serv*.mp.  model of care.mp.  delivery of care.mp.  medical home care.mp.  integr* care.mp.  integrated care*  emergency admission*.mp.  school*.mp.  wrap-around care*.mp.  emergency readmission*.mp.  wrap-around care*.mp.  rehospitali*.mp.  (hospital adj3 emergenc*).mp.  (emergenc* adj2 medic*).mp.  acute hospital*.mp.  (acute adj2 medic*).mp.  (emergenc* adj2 treatment*).mp.  re?feeding.mp.  (emergenc* adj2 in?patient).mp.  (non?elective adj2 care).mp.  (non?elective adj2 treatment).mp.  (unscheduled adj2 care).mp.  (unscheduled adj2 treatment).mp.  (unscheduled adj2 medical).mp.  (non?elective adj2 medical).mp.  (unplanned adj2 care).mp.  (unplanned adj2 treatment).mp.  (unplanned adj2 medical).mp.  (prospective adj2 care).mp.  (prospective adj2 treatment).mp.  (prospective adj2 medical).mp.  (urgent adj2 care).mp.  (urgent adj2 treatment).mp.  (urgent adj2 medical).mp.  emergency hospitali?ation*.mp.  acute hospitali?ation.mp.  acute treatment*.mp.  acute admission*.mp. | brief intiv*.mp.  crisis interven*.mp.  famil*.mp.  cognitive* behav*.mp.  dialect* behav*.mp.  well*.mp.  counsel*.mp.  family therapy.mp.  individ*consel*.mp.  psycho* therapy*.mp.  parent-child dyad  healthcare* deliv*.mp.  invidi* counsel*.mp  parent*therap*.mp.  school based therp*.mp.  psycho care.mp.  group session*.mp.  group thera*.mp.  counsel*.mp.  care co-ordin*.mp.  home* care.mp.  patient cent* care.mp.  community based care.mp.  holistic care.mp.  safety care.mp.  risk assessmen*.mp.  In hospital*.mp.  drug counsel*.mp.  pharmacotherapy*.mp.  medication manage*.mp.  assessment serv*.mp.  model of care.mp.  delivery of care.mp.  medical home care.mp.  integr* care.mp.  integrated care*  emergency admission*.mp.  school*.mp.  wrap-around care*.mp.  emergency medicine/ or pediatric emergency medicine/  acute care.mp.  acute ward.mp.  emergency services/ or crisis intervention services/or emergency medicine/  hospital emergency service/ or emergency health service/  emergency medicine/  emergency treatment/ | brief intiv*.mp.  crisis interven*.mp.  famil*.mp.  cognitive* behav*.mp.  dialect* behav*.mp.  well*.mp.  counsel*.mp.  family therapy.mp.  individ*consel*.mp.  psycho* therapy*.mp.  parent-child dyad  healthcare* deliv*.mp.  invidi* counsel*.mp  parent*therap*.mp.  school based therp*.mp.  psycho care.mp.  group session*.mp.  group thera*.mp.  counsel*.mp.  care co-ordin*.mp.  home* care.mp.  patient cent* care.mp.  community based care.mp.  holistic care.mp.  safety care.mp.  risk assessmen*.mp.  In hospital*.mp.  drug counsel*.mp.  pharmacotherapy*.mp.  medication manage*.mp.  assessment serv*.mp.  model of care.mp.  delivery of care.mp.  medical home care.mp.  integr* care.mp.  integrated care*  emergency admission*.mp.  school*.mp.  wrap-around care*.mp.  emergency admission*.mp.  wrap-around care*.mp.  emergency readmission*.mp.  rehospitali*.mp.  (hospital* adj4 emergenc*).mp.  (emergenc* adj2 medic*).mp.  acute hospital*.mp.  (acute adj2 medic*).mp.  (emergenc* adj2 treatment*).mp.  medical stabili?ation.mp  re?feeding.mp.  (acute adj2 in?patient*).mp.  (emergenc* adj2 in?patient*).mp.  (hospital adj2 readmi*).mp.  (non?elective adj2 care).mp.  (non?elective adj2 admission*).mp.  (non?elective adj2 treatment).mp.  (non?elective adj2 readmission*).mp.  (unscheduled adj2 care).mp.  (unscheduled adj2 admission*).mp.  (unscheduled adj2 readmission*).mp.  (unscheduled adj2 treatment).mp.  (unscheduled adj2 hospital*).mp.  (unplanned adj2 hospital*).mp.  (unplanned adj2 admi*).mp.  (unplanned adj2 care).mp.  (unplanned adj2 treatment).mp.  (unplanned adj2 readmi*).mp.  (non?elective adj2 hospital*).mp  (prospective adj2 care).mp.  (prospective adj2 admission*).mp.  (prospective adj2 treatment).mp.  (prospective adj2 readmission*).mp.  (urgent adj2 care).mp.  (urgent adj2 admission*).mp.  (urgent adj2 readmission*).mp.  (urgent adj2 treatment).mp. | brief intiv*.mp.  crisis interven*.mp.  famil*.mp.  cognitive* behav*.mp.  dialect* behav*.mp.  well*.mp.  counsel*.mp.  family therapy.mp.  individ*consel*.mp.  psycho* therapy*.mp.  parent-child dyad  healthcare* deliv*.mp.  invidi* counsel*.mp  parent*therap*.mp.  school based therp*.mp.  psycho care.mp.  group session*.mp.  group thera*.mp.  counsel*.mp.  care co-ordin*.mp.  home* care.mp.  patient cent* care.mp.  community based care.mp.  holistic care.mp.  safety care.mp.  risk assessmen*.mp.  In hospital*.mp.  drug counsel*.mp.  pharmacotherapy*.mp.  medication manage*.mp.  assessment serv*.mp.  model of care.mp.  delivery of care.mp.  medical home care.mp.  integr* care.mp.  integrated care*  emergency admission*.mp.  school*.mp.  wrap-around care*.mp.  emergency medicine/ or pediatric emergency medicine/  well*.mp.  counsel*.mp.  family therapy.mp.  individ*consel*.mp.  psycho* therapy*.mp.  healthcare* deliv*.mp.  parent-child dyad  acute care.mp.  Emergency Service, Hospital  Child, Hospitalized/ or Adolescent, Hospitalized/  hospitalisation/ or “length of stay” / or patient admission/ or patient readmission | brief intiv*.mp.  crisis interven*.mp.  famil*.mp.  cognitive* behav*.mp.  dialect* behav*.mp.  well*.mp.  counsel*.mp.  family therapy.mp.  individ*consel*.mp.  psycho* therapy*.mp.  parent-child dyad  healthcare* deliv*.mp.  invidi* counsel*.mp  parent*therap*.mp.  school based therp*.mp.  psycho care.mp.  group session*.mp.  group thera*.mp.  counsel*.mp.  care co-ordin*.mp.  home* care.mp.  patient cent* care.mp.  community based care.mp.  holistic care.mp.  safety care.mp.  risk assessmen*.mp.  In hospital*.mp.  drug counsel*.mp.  pharmacotherapy*.mp.  medication manage*.mp.  assessment serv*.mp.  model of care.mp.  delivery of care.mp.  medical home care.mp.  integr* care.mp.  integrated care*  emergency admission*.mp.  school*.mp.  wrap-around care*.mp.  hospital near/2 emergenc*  healthcare* deliv*.mp.  well*.mp.  counsel*.mp.  family therapy.mp.  individ*consel*.mp.  psycho* therapy*.mp.  parent-child dyad  emergenc* near/1 medic*  “acute hospital*”  “emergency service, hospital”  “emergency medicine”  “pediatric emergency medicine”  emergenc* near/1 treatment*  “medical stabil?ation”  refeeding  emergenc* near/1 inpatient*  nonelective near/1 care  nonelective near/1 treatment  unscheduled near/1 medical  nonelective near/1 medical  unplanned near/1 care  unplanned near/1 treatment  acute near/1 medic*  unscheduled near/1 care  unscheduled near/1 treatment  unplanned near/1 medical  prospective near/1 care  prospective near/1 treatment  prospective near/1 medical  urgent near/1 care  urgent near/1 treatment  urgent near/1 medical  hospitali*  length of stay near/2 acute  acute near/1 inpatient*  hospital near/1 readmi*  nonelective near/1 admission*  nonelective near/1 readmission*  unscheduled near/1 admission*  unscheduled near/1 readmission*  unscheduled near/1 hospital*  unplanned near/1 hospital*  unplanned near/1 admi*  unplanned near/1 readmi*  nonelective near/1 hospital*  Child, Hospitalised  Adolescent, Hospitalised  prospective near/1 admission*  prospective near/1 readmission*  urgent near/1 admission*  urgent near/1 readmission*  “emergency hospitalisation*”  “emergency hospitalization*”  “acute hospitalisation*”  “acute hospitalization*”  “acute treatment*”  “acute admission*”  “acute ward”  “emergency services”  “crisis intervention services” | brief intiv*.mp.  crisis interven*.mp.  famil*.mp.  cognitive* behav*.mp.  dialect* behav*.mp.  well*.mp.  counsel*.mp.  family therapy.mp.  individ*consel*.mp.  psycho* therapy*.mp.  parent-child dyad  healthcare* deliv*.mp.  invidi* counsel*.mp  parent*therap*.mp.  school based therp*.mp.  psycho care.mp.  group session*.mp.  group thera*.mp.  counsel*.mp.  care co-ordin*.mp.  home* care.mp.  patient cent* care.mp.  community based care.mp.  holistic care.mp.  safety care.mp.  risk assessmen*.mp.  In hospital*.mp.  drug counsel*.mp.  pharmacotherapy*.mp.  medication manage*.mp.  assessment serv*.mp.  model of care.mp.  delivery of care.mp.  medical home care.mp.  integr* care.mp.  integrated care*  emergency admission*.mp.  school*.mp.  wrap-around care*.mp.  “acute care”  “emergency admission*”  school*.mp.  “emergency readmission*”  rehospitali*  hospital near/2 emergenc*  wrap-around care*.mp.  healthcare* deliv*.mp.  emergenc* near/1 medic*  well*.mp.  counsel*.mp.  family therapy.mp.  individ*consel*.mp.  psycho* therapy*.mp.  parent-child dyad  “acute hospital*”  “emergency service, hospital”  “emergency medicine”  “pediatric emergency medicine”  emergenc* near/1 treatment*  “medical stabil?ation”  refeeding  emergenc* near/1 inpatient*  nonelective near/1 care  nonelective near/1 treatment  unscheduled near/1 medical  nonelective near/1 medical  unplanned near/1 care  unplanned near/1 treatment  acute near/1 medic*  unscheduled near/1 care  unscheduled near/1 treatment  unplanned near/1 medical  prospective near/1 care  prospective near/1 treatment  prospective near/1 medical  urgent near/1 care  urgent near/1 treatment  urgent near/1 medical  hospitali*  length of stay near/2 acute  acute near/1 inpatient*  hospital near/1 readmi*  nonelective near/1 admission*  nonelective near/1 readmission*  unscheduled near/1 admission*  unscheduled near/1 readmission*  unscheduled near/1 hospital*  unplanned near/1 hospital*  unplanned near/1 admi*  unplanned near/1 readmi*  nonelective near/1 hospital*  Child, Hospitalised  Adolescent, Hospitalised  prospective near/1 admission*  prospective near/1 readmission*  urgent near/1 admission*  urgent near/1 readmission*  “emergency hospitalisation*”  “emergency hospitalization*”  “acute hospitalisation*”  “acute hospitalization*”  “acute treatment*”  “acute admission*”  “acute ward”  “emergency services”  “crisis intervention services” |
